# Frost trends and their estimated impact on yield in the Australian wheatbelt

**DOI:** 10.1093/jxb/erv163

**Published:** 2015-04-28

**Authors:** Bangyou Zheng, Scott C. Chapman, Jack T. Christopher, Troy M. Frederiks, Karine Chenu

**Affiliations:** ^1^CSIRO Agriculture Flagship, Queensland Bioscience Precinct, 306 Carmody Road, St Lucia, QLD 4067, Australia; ^2^The University of Queensland, Queensland Alliance for Agriculture and Food Innovation (QAAFI), Leslie Research Facility, PO Box 2282 Toowoomba, QLD 4350, Australia; ^3^Queensland Department of Agriculture, Fisheries and Forestry (DAFFQ), Leslie Research Facility, PO Box 2282 Toowoomba, QLD 4350, Australia; ^4^The University of Queensland, Queensland Alliance for Agriculture and Food Innovation (QAAFI), 203 Tor Street, Toowoomba, QLD 4350, Australia

**Keywords:** Breeding, climate change, crop adaptation, crop modelling, ideotype, post-head-emergence frost, reproductive frost, spring radiant frost.

## Abstract

Radiant spring frosts occurring during reproductive developmental stages can result in catastrophic yield loss for wheat producers. To better understand the spatial and temporal variability of frost, the occurrence and impact of frost events on rain-fed wheat production was estimated across the Australian wheatbelt for 1957–2013 using a 0.05 ° gridded weather data set. Simulated yield outcomes at 60 key locations were compared with those for virtual genotypes with different levels of frost tolerance. Over the last six decades, more frost events, later last frost day, and a significant increase in frost impact on yield were found in certain regions of the Australian wheatbelt, in particular in the South-East and West. Increasing trends in frost-related yield losses were simulated in regions where no significant trend of frost occurrence was observed, due to higher mean temperatures accelerating crop development and causing sensitive post-heading stages to occur earlier, during the frost risk period. Simulations indicated that with frost-tolerant lines the mean national yield could be improved by up to 20% through (i) reduced frost damage (~10% improvement) and (ii) the ability to use earlier sowing dates (adding a further 10% improvement). In the simulations, genotypes with an improved frost tolerance to temperatures 1 °C lower than the current 0 °C reference provided substantial benefit in most cropping regions, while greater tolerance (to 3 °C lower temperatures) brought further benefits in the East. The results indicate that breeding for improved reproductive frost tolerance should remain a priority for the Australian wheat industry, despite warming climates.

## Highlight

Over the last decades, the impact of post-heading frost on yield has increased in major parts of the Australian wheatbelt. Despite global warming, frost remains a high priority for breeding.

## Introduction

Post-head-emergence frosts (PHEF) are catastrophic in wheat, with a single frost event having the potential to devastate individual crops by damaging stems and killing whole heads (Frederiks *et al.*, 2012). PHEFs are common in subtropical areas, but can also occur in Mediterranean and temperate regions, including South America, Canada, Russia, the USA, and Australia (Al-Khatib and Paulsen, 1984; Boer *et al.*, 1993; Fuller *et al.*, 2007). Regional PHEF yield penalties of 10% commonly occur, but losses in excess of 85% have also been observed in certain seasons in the USA and Australia (Paulsen and Heyne, 1983; Boer *et al.*, 1993).

In Australia, ‘spring wheat’ is typically planted in autumn, growing through winter, and harvested in spring. Although diurnal temperature variation is generally too high to allow cold acclimation, significant vegetative-frost damage remains infrequent in the Australian wheatbelt (Single, 1991; Woodruff *et al.*, 1997). When vegetative damage occurs, young crops usually regrow from superficial leaf scorching, particularly when moisture is available (Afanasev, 1966). Before the head emerges, the fragile reproductive structures are partially protected from frost by the flag leaf sheath, which reduces damage and subsequent yield losses (Fuller *et al.*, 2007). Sensitivity to frost increases sharply after the awns or spikes start to emerge from the auricle of the flag leaf (Livingston and Swinbank, 1950; Single, 1964; Afanasev, 1966; Paulsen and Heyne, 1983). Overall, wheat crops are most sensitive after head emergence (‘heading’). Thus, where frost risk is high, management of crop phenology is necessary to avoid post-heading frosts and maintain an acceptable frost risk (Frederiks *et al.*, 2004).

Over the last century, mean temperatures in Australia have been increasing on average by 0.09 °C per decade (Murphy and Timbal, 2008). While frost events vary spatially and from season to season across the wheatbelt (Stone *et al.*, 1996; Alexander *et al.*, 2006; Crimp *et al.*, 2015), more hot days and fewer cold days are predicted for future climates (Stone *et al.*, 1996; Collins *et al.*, 2000). However, while slightly counter-intuitive, global warming may increase the risk of frost by (i) accelerating wheat phenology, so that heading time occurs earlier in spring; or (ii) increasing the frequency of clear nights during drought (Gu *et al.*, 2008; Zheng *et al.*, 2012).

In PHEF-prone regions, wheat producers manage frost risk by adapting sowing time and variety. Models that estimate the timing of sensitive growth stages have long been used in Australia to assist in determining frost risks associated with different management decisions (e.g. Hammer *et al.*, 1987; Woodruff, 1992; Farre *et al.*, 2010; Bell *et al.*, 2015). Recently, Zheng *et al.* (2013) successfully tested a model of heading time across the Australian wheatbelt (4475 observations) that includes effects of the major vernalization (*VRN1*) and photoperiod (*Ppd-D1*) genes with a residual mean squared error (RSME) of only 4.3 d. Using historical (and predicted future) climate data, these models can estimate heading or flowering dates for combinations of sowing time and variety, and provide a powerful tool for producers to reduce frost risk.

Current elite wheat cultivars are sensitive to post-heading frosts, which constrain sowing time flexibility and variety choice. Some variations to PHEF tolerance have been reported in barley (Reinheimer *et al.*, 2004; Chen *et al.*, 2009*a*, *b*; Frederiks *et al.*, 2011). However, despite active screening for PHEF tolerance, no wheat lines with tolerance greater than current cultivars have been identified (Frederiks *et al.*, 2012).

Breeding for improved PHEF tolerance would allow greater yield to be achieved, as (i) direct frost damage could be reduced and (ii) crops could potentially be sown earlier to reduce risks of late-season drought and/or heat stresses. Substantial increases in yield in the order of 30–50% have been observed in Australian PHEF-prone regions in seasons when early flowering cereal crops managed to escape frost (Frederiks *et al.*, 2011). In Queensland, Woodruff and Tonks (1983) showed that maximum yields were associated with mid-winter flowering time, as long as frost did not occur. Yield reductions of up to 16% per week of flowering delay past the optimum time have been estimated for wheat and canola in Australia (Doyle and Marcellos, 1974; Marcellos and Doyle, 1974; Woodruff and Tonks, 1983; Robertson *et al.*, 1999). This reduction in yield and yield potential is associated both with accelerated development, which reduces the vegetative and grain-filling periods, and with increased terminal drought as the soil water storage becomes depleted.

The aims of this study were to (i) characterize climatic trends in frost events in the last six decades; and (ii) estimate the comparative benefits that breeding for PHEF tolerance would bring to the wheat industry in Australia. To the authors’ knowledge, no systematic record of the frequency, intensity, or estimated yield loss due to PHEF has been reported for Australia or elsewhere. Growers do not commonly keep detailed records of PHEF damage and it is difficult to estimate the level of yield that may have been achieved if frost had not occurred. Here, the trends of frost occurrence were analysed and frost impacts were estimated for early-, mid-, and late-maturing wheat cultivars across the Australian wheatbelt using gridded and point-based historical climate data sets. Given the importance of accurate phenology prediction to estimate frost impacts, a widely tested gene-based model of APSIM-Wheat was used to predict wheat heading times (Zheng *et al.*, 2013). To evaluate the potential comparative benefits from different levels of genetic adaptation, yield gains for improved levels of frost tolerance were assessed by modelling the effects of a range of damage threshold temperatures.

## Materials and methods

### Climatic data

**Fig. 1.**
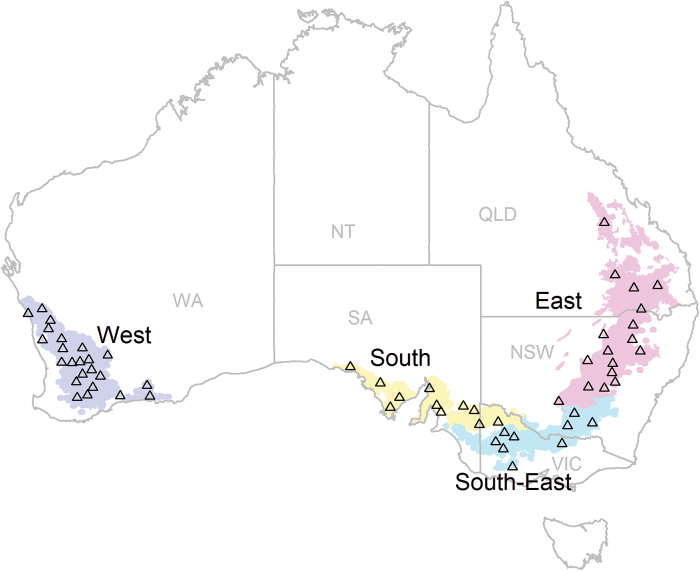
Map of 60 representative sites of the Australian wheatbelt. The wheatbelt is represented by four regions: East (pink), South-East (turquoise), South (yellow), and West (dark blue). The abbreviations in grey correspond to the Australian states of Queensland (QLD), New South Wales (NSW), Victoria (VIC), South Australia (SA), and Western Australia (WA) as well as the Northern Territory (NT). See Chenu *et al.* (2013) for a detailed list of locations (longitude and latitude), soil characteristics, initial soil water, and fertilization used in the simulations.

Frost trends and direct impacts were calculated for spatial and temporal scales across the Australian wheatbelt (Fig. 1) using daily climatic data from 1957 to 2013 from the SILO data drill data set (Jeffrey *et al.*, 2001) with a spatial resolution of 0.05 °×0.05 ° (~ 5 km×5 km).

To investigate the potential yield benefits of improved frost tolerance, including associated benefits related to earlier sowing dates, 60 sites were selected across the Australian wheatbelt (Fig. 1), each representing a similar average annual area planted to wheat as described by Chenu *et al.* (2013). Daily data from the 60 weather stations were extracted from the SILO patched point data set (Jeffrey *et al.*, 2001).

### Crop simulations

Yield and Zadoks decimal phenological stages (Zadoks *et al.*, 1974) were simulated for the 60 sites using the APSIM 7.6 model (Holzworth *et al.*, 2014) with a wheat phenology gene-based module (Zheng *et al.*, 2013). Frost impacts were modelled as detailed below. Simulations were conducted for wheat crops sown at 1 d intervals within a fixed sowing window from 1 May to 21 June for all 60 sites, except for Emerald, in the north-east of the wheatbelt, where sowing was simulated from 15 April to 7 June to reflect common local farming practices (Supplementary Table S1 available at *JXB* online; Chenu *et al.*, 2013). Nitrogen fertilization was simulated so as to reflect local farming practices and therefore varied with location and seasonal rainfall (Supplementary Table S1; Chenu *et al.*, 2013). Soil water contents at sowing were set to five levels each representing 20% of long-term conditions encountered for each site, thus considering each season independently from previous seasons (Supplementary Table S1; Chenu *et al.*, 2013). In the analysis, yield from crops sown at the same site and on the same date were averaged across the five levels of initial soil water. For each of the four main regions of the wheatbelt (‘East’, ‘South’, ‘South-East’, and ‘West’; Fig. 1), three cultivars were chosen to represent locally grown early-, mid-, and late-maturing cultivars (Table 1).

**Table 1.** Cultivars chosen to represent early-, mid-, and late- maturing cultivars for each region

Region	Early-maturing	Mid-maturing	Late-maturing
East	Ventura	Baxter	Sunbri
South	Axe	Janz	Bolac
South-East	Axe	Janz	Bolac
West	Westonia	Mace	Yitpi

### Estimation of frost impact on yield

Frost susceptibility varies with growth stage. Wheat is most frost tolerant in the vegetative stages, with susceptibility increasing with plant maturity. In the model, no vegetative-frost impact was considered, as some preliminary simulations of frost effects revealed no substantial effect on yield given the low frequency of damaging early frosts (data not shown).

Wheat becomes more susceptible to frost when the spike emerges from the flag leaf sheath (i.e. first awns visible, Zadoks stage Z49; Single, 1964). Sensitivity to frost increases after the awns or spikes start to emerge from the flag leaf (Livingston and Swinbank, 1950; Single, 1964; Afanasev, 1966; Paulsen and Heyne, 1983). In the model, post-heading frost was estimated at the field level, and the plant phenology was simulated for average growing stages. However, in reality, spikes of different tiller cohorts emerge both before and after the field average reaches Z49. To approximate the distributions of exposed heads at susceptible post-heading stages, a multiplier was applied from 1 (i.e. no yield loss) at the late-booting average stage (Z45) followed by a linear decrease to 0.1 (i.e. 90% yield loss) against Zadoks score up to mid-heading (Z55), when almost all tillers would have reached the susceptible post-heading stage (Z49). Maximum susceptibility (i.e. all tillers susceptible) was then maintained until the start of dough development (Z80), with a constant yield multiplier of 0.1 (i.e. 90% yield loss) over the developmental period Z49–Z80 for each day with a minimum temperature below a threshold of 0 °C. After Z80, the yield multiplier was linearly increased over time (from 0.1 to 1) up to the completion of dough development (Z89), which reflected late frost events causing cessation of grain development.

Climatic data used to determine frost occurrence were measured within a Stevenson screen as these are the only reliable source of long-term temperature records for the Australian wheatbelt. However, Stevenson screen measurements are typically several degrees higher that the temperatures of the crop canopy during radiant frost events (Marcellos and Single, 1975; Frederiks *et al.*, 2011, 2012). Previous studies have shown that after heading, crops are only damaged by canopy temperatures several degrees below 0 °C (Single, 1985; Frederiks *et al.*, 2012). To determine a Stevenson screen temperature threshold, temperatures from –5 °C to +2 °C were assessed in 1 °C increments (data not shown). Based on consultation with agronomists and on trial data, it was determined that a threshold temperature of 0 °C best explained major recent incidences of frost damage. Using this threshold, simulations predicted that heading would occur after the main, mid-winter frost risk period when sowing dates recommended by industry guidelines were used for known frost-prone areas (Hollaway, 2014; Lush, 2014; Matthews *et al.*, 2014; Shackley *et al.*, 2014; Wheeler, 2014). Hence, a 0 °C threshold was used in the model-based simulations (‘Control’ or ‘Ctrl’).

### Simulation of different frost-tolerance levels

Current elite Australian wheat varieties were considered to be affected by post-heading Stevenson screen temperature below the 0 °C threshold as described above. To estimate the potential benefit of genotypes with improved tolerance, the simulations were conducted for a range of damage threshold temperatures from –1 °C to –5 °C (FT_1_ to FT_5_). Total frost tolerance (FT_tot_) was also simulated, representing a virtual genotype that is insensitive to frosts of any temperature.

### Direct and indirect impacts of frost

For each level of frost tolerance (FT_1–5_ and FT_tot_), two types of impact were estimated: (i) a direct impact reflecting the direct frost damage with no change in management; and (ii) a direct plus indirect impact where both the direct frost damage and the indirect effects from adaptation of sowing date to the new levels of frost tolerance were considered. For each location×cultivar×sowing date (sowing at 1 d interval) combination, an average yield was calculated for the 1957–2013 period. The optimum sowing day corresponding to the maximum simulated mean yield (1957–2013) was identified for the Control early-, mid-, and late-maturing cultivars (threshold of 0 °C) and the frost-tolerant early-, mid-, and late-maturing virtual genotypes (thresholds below 0 °C).

For each maturity type, direct impact on yield was investigated by comparing the yield for (i) the control (frost-tolerance of 0 °C) and (ii) each of the virtual genotypes with different levels of frost tolerance (FT_1–5_ and FT_tot_) with all crops sown at the optimum sowing date of the Control (i.e. no change in management). For the direct plus indirect frost impact, optimum yield of frost-tolerant virtual genotypes (FT_1–5_ and FT_tot_) was calculated by re-estimating the optimum sowing date of each genotype, while considering their respective levels of frost tolerance.

### Average occurrence and trends of frost events and impacts

To calculate the trend of frost events since 1957, multiple thresholds of minimum air temperature (*T*_min_) from 0 °C to –5 °C were considered. For each of the locations (grid data at 0.05 °), the number of ‘frost’ days was defined as the average for 1957–2013 of the annual number of days with a minimum temperature below the considered threshold. The last frost day of each year was defined as the last day of the year with a *T*_min_ below the considered threshold. The last frost day for each location was calculated as the 90th percentile of last frost days for 1957–2013; that is, when there is a <10% chance for a ‘frost’ to occur later than this date (Reyenga *et al.*, 1999; Zheng *et al.*, 2012). Herein, so-called ‘frost-free’ regions refer to grid points where fewer than 10% of years were affected by ‘frost’ events at any time.

Temporal trends of last frost days, number of frost days, and frost impacts were calculated using least squares linear regression on annual data from 1957 to 2013 for each spatial cell (0.05 °×0.05 °). The significance of trends was estimated following a method that considered the temporal autocorrelation by reducing the effective sample size of the time series (Santer *et al.*, 2000).

All maps and statistical analyses were generated using the R language Version 3.0 (R Development Core Team, 2014). Significance of temporal trends were tested for *P*<0.1, as in Dobrowski *et al.* (2013).

## Results

### Frost events occur in most of the wheatbelt

**Fig. 2.**
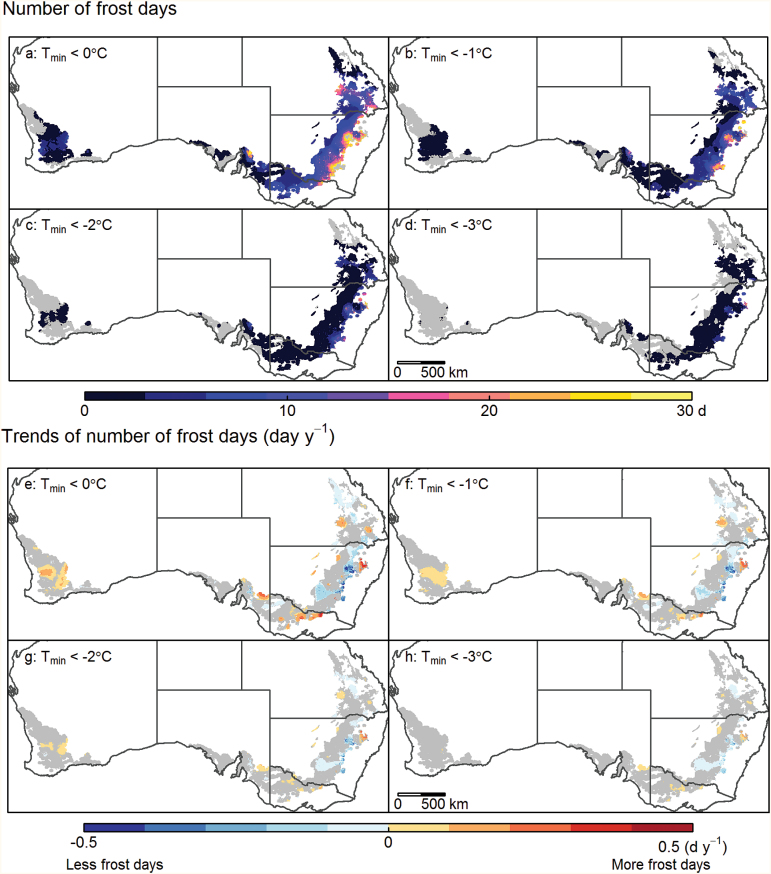
Maps of the average annual number of frost days over the 57 years from 1957 to 2013 (a–d) and the temporal trend of number of frost days (e–h) for minimum temperature (*T*_min_) thresholds of 0 °C (a and e), –1 °C (b and f), –2 °C (c and g), and –3 °C (d and h) across the Australian wheatbelt. The grey shading in (a–d) indicates ‘frost-free’ regions where frost at the specified temperature threshold occurred in <10% of years and in (e–h) regions where trends in number of frost days were not significant (*P*>0.1) or for which there were <10% years with frost.

Frost can occur in most parts of the Australian wheatbelt at times which potentially correspond to the post-heading stage in wheat, for example August–September (Fig. 2a). However, a number of virtually ‘frost-free’ regions exhibited frost (*T*_min_<0 °C) occurrence in fewer than 10% of years (grey areas, Fig. 2a). Frost-free areas are found in the northern, western, and southern parts of the West region, coastal areas of the South region, and northern most areas of the East region (Dawson-Callide valley of Queensland). Significant areas of the wheatbelt have an average of 6–12 frost days per annum, including a large proportion of the Eastern and Southern wheatbelt (blue areas, Fig 2a). Areas with >12 frost days occurred in the Eastern wheatbelt (Burnett, eastern Darling Downs, southern Queensland, and more elevated areas of New South Wales; purple areas, Fig 2a). Severely frost-prone areas with ≥20 frost days per year on average occurred at higher altitudes, where only small areas of wheat are grown (pink and yellow areas, Fig 2a). Overall, most of the wheatbelt had ≤12 frost days (<0 °C) in an average year.

**Fig. 3.**
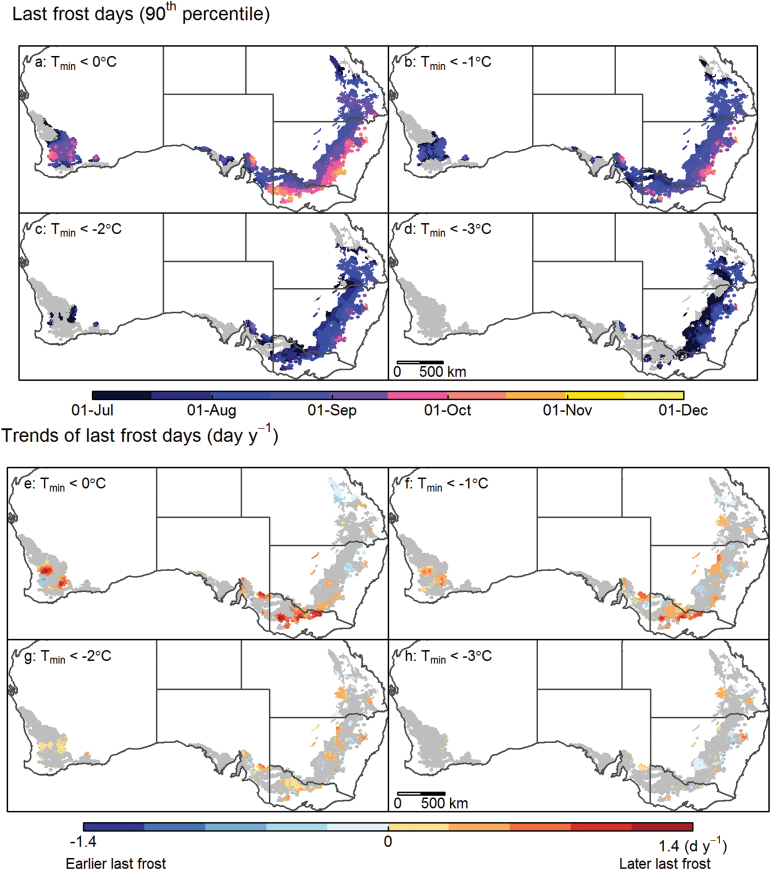
Maps of the last frost day of the year (90th percentile; a–d) and the temporal trend of last frost day (e–h) for minimum temperature (*T*_min_) thresholds of 0 °C (a and e), –1 °C (b and f), –2 °C (c and e), and –3 °C (d and h) across the Australian wheatbelt. The grey shading in (a–d) indicates ‘frost-free’ regions where ‘frost’ events at the specified temperature threshold occurred in <10% of years, and in (e–h) regions where trends in last frost days were not significant (*P*>0.1) or for which there were <10% years with frost. Data correspond to the last frost day over 57 years (1957–2013).

To understand how improving genotypic frost tolerance by a few degrees would impact spatially, maps were produced for different temperature thresholds (Figs 2, 3). For a threshold of –2 °C (Fig. 2c), most parts of the West and South regions would be considered ‘frost’ free, with only small pockets of ‘frost’ risk area for this temperature. In contrast, severe ‘frost’ risks remained in the majority of areas in the East and South-East, with the exception of the most northern part of the wheatbelt. The risk of ‘frost’ occurrence also greatly decreased when considering a –2 °C rather than a 0 °C threshold, and most of the wheatbelt had fewer than 3 d with *T*_min_< –2 °C in average years, except in some areas of the East (Fig. 2c).

Industry advisors and grain producers generally try to avoid frost risks in >10% of years. Wheat crops are thus typically sown at a date that will ensure heading occurs after what is referred to here as the ‘last frost day’ (i.e. when frost is predicted in <10% of seasons). Depending on the location and the temperature threshold considered, the last frost day ranged from July to December (Fig. 3a–d), thus potentially severely affecting wheat yield. In general, areas with more frost days tended to have lower seasonal minimum temperature and to exhibit a later ‘last frost day’ (Fig. 2a; Supplementary Figs S2a, 3a at *JXB* online). Similarly, the spatial distribution for ‘last frost day’ showed a similar trend to that for seasonal minimum temperature and that for number of frost days (Fig. 2e; Supplementary Figs S2b, 3e). The number of frost days and the date of the last frost day correlated within each region and each temperature threshold (*r*=0.52–0.90 for 0 °C; Figs 2, 3; Supplementary Fig. S1). Overall, the probability of frosts (*T*_min_<0 °C) dropped to <10% by the first week of September, or earlier, in most of the Australian wheatbelt.

### Trends in frost occurrence since 1957 vary across the Australian wheatbelt

Since 1957, large areas showed no significant trend for the number of frost days (*P*>0.1; grey areas Fig. 2e, f). However, a significantly increased number of frost events (*P*<0.1) has been recorded for several areas within each region of the wheatbelt (Fig. 2e). For instance, up to an extra 0.5 frost day per year has been recorded on average in higher altitude areas. In contrast, significant decreases in frost occurrence were observed across almost half of the East region: as much as –0.5 frost day per year in some areas (Fig. 2e). Similar but weaker trends were observed for other threshold temperatures (–1, –2, and –3 °C; Fig. 2f–h). In general, areas with an increasing number of frost days at 0 °C tended also to have an increasing number of days (Fig. 2f–h) at lower thresholds.

As for the number of frost days, ‘last frost days’ showed no significant change (*P*>0.1) in a large part of the wheatbelt over the last six decades (grey areas, Fig. 3e, f). However, significant delays in last frost days (*P*<0.1) were recorded particularly in the South, South-East, and West regions, with shifts up to 1.4 d year^–1^ later occurring in the South and West. That is, the last frost day has recently been as much as 80 d later than it was in 1957 (Fig. 3e, f). In contrast, both earlier and later occurrences of the last frost day were recorded in the East (Fig. 3e–h). Overall, observed temporal trends tended to weaken with lower temperature thresholds (Fig. 3e–h).

### Frost limits achievable yield in most of the Australian wheatbelt

**Fig. 4.**
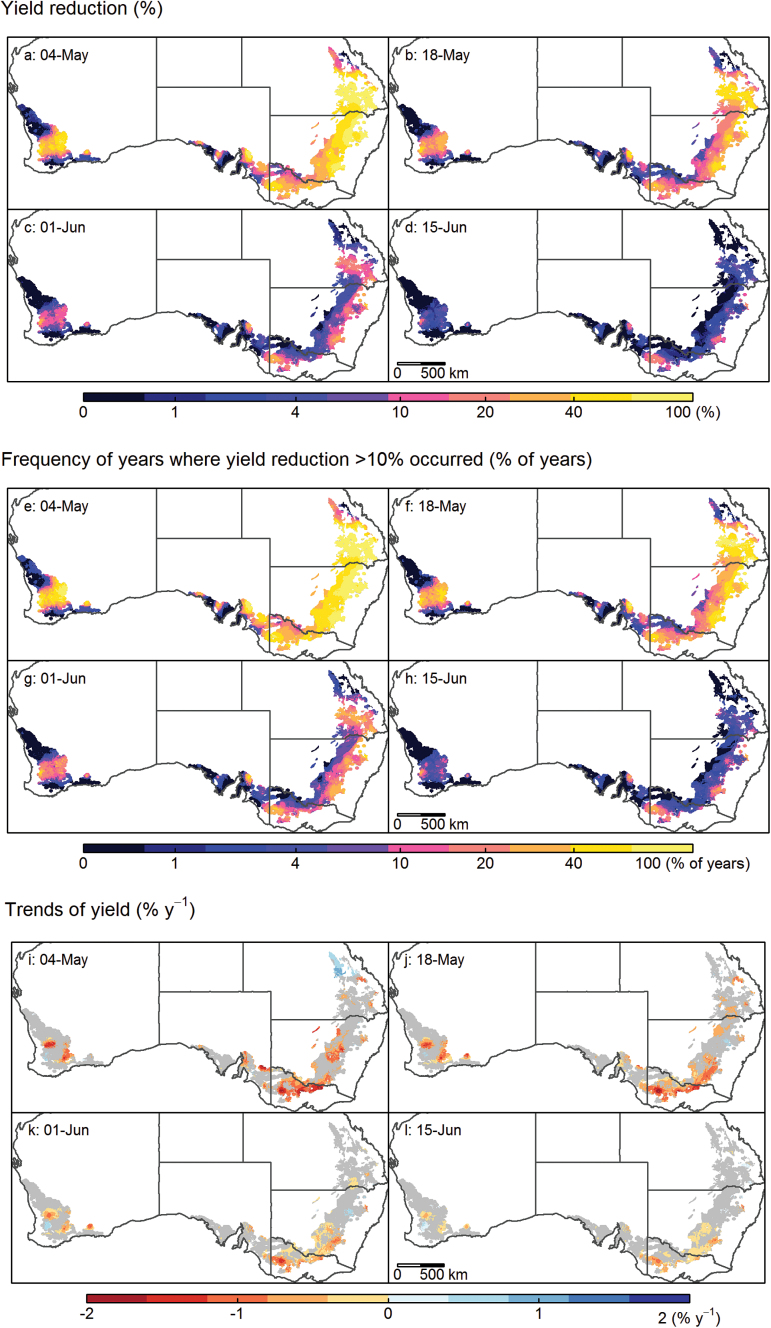
Maps for the mid-maturity cultivar Janz of average simulated yield reduction due to frost (*T*_min_<0 °C) in the 57 years from 1957 to 2013 (a–d), the frequency of years (% of years) when yield reduction <10% occurred (e–h), and trends over time in yield (i–l) for sowing at 4 May (a, e, and i), 18 May (b, f, and j), 1 June (c, g, and k) and 15 June (d, h, and i) across the Australian wheatbelt. See also Supplementary Figs S2–S4 at *JXB* online for other *T*_min_ thresholds and genotypes of different phenology classes.

As expected from the variations observed in occurrence of frost days, minimum temperatures, and last frost days (Figs 2, 3; Supplementary Fig. S2 at *JXB* online), high spatial heterogeneity was simulated for average annual yield loss over the 1957–2013 period (Fig. 4a). Near total crop losses in most years were predicted for a mid-maturing cultivar Janz sown in early May over large areas of the East and South and inland parts of the West. This result concurs with local agronomic advice which does not recommend such early sowing of a mid-maturing cultivar. Frost impacts were reduced for later sowing, from mid-May onwards. For the mid-maturity cultivar Janz grown in the East or in the middle part of the West, a high risk of ≥10% yield reduction was simulated for plantings in early to mid-May (Fig. 4e, f). Low risks of yield reduction only occurred for most parts of the wheatbelt when sowing was delayed until early to mid-June (Fig. 4g, h). Overall, the lowest average crop losses were simulated for the northern parts of the West region, close to the Great Australian Bight in the South and West regions, coastal areas of the South, and in the most northern part of the East region.

As anticipated, cultivars that flower early were even more prone to frost than mid-maturity lines when sown in early May (Supplementary Fig. S4 at *JXB* online). In some frost-prone areas, sowing later than mid-June was required to reduce the average yield loss to <10% for early cultivars (Supplementary Fig. S4d). Later sowing and longer season cultivars led to lower yield reduction (Fig. 4; Supplementary Figs S3–S5), as crops escape the main mid-winter frost-risk period. Even with late cultivars such as Sunbri, the average yield reduction remained high for early May sowings for many areas (Supplementary Fig. S5a). Low risks of yield reduction were achieved by Sunbri from mid-May onwards (Supplementary Fig. S5b–d).

Trends over time revealed that simulated yield did not vary significantly (*P*>0.1) since 1957 in half or more of the wheatbelt (Fig. 4), regardless of the maturity class (Fig. 4; Supplementary Figs S4, S5 at *JXB* online). Significant yield increases up to 0.8% of yield per year were simulated in small parts of the Australian wheatbelt where fewer frost events occurred in recent years, such as the most northern part of the East and a small part of the central West, especially for earlier sowings (*P*<0.1, Figs 2e, 4i–l). In contrast, greater frost damage of as much as –1.5% of yield per year (i.e. 85% since 1957) was simulated in parts of the wheatbelt such as in the South-East and West, especially for earlier sowings (*P*<0.1; Fig. 4i–l). Overall, simulations suggest that a larger cropping area has been affected by greater yield loss over the last six decades (Fig. 4i–l; Supplementary Figs S4i–l, S5i–l) due to more frost days (Fig. 2e) and/or a delay in last frost day (Fig. 3e).

### Potential benefits of frost-tolerant genotypes differ between the East and West

Currently, reducing frost impact on wheat yield in frost-prone regions of Australia is done by adapting the sowing time to ensure that heading occurs after the last frost day. However, later sowing increases the risk of terminal drought and heat stress during grain filling, and thus reduces yield potential. Sowing windows are thus typically determined for each variety to limit abiotic risks such as frost, heat, and drought (e.g. Supplementary Fig. S6 at *JXB* online).

**Fig. 5.**
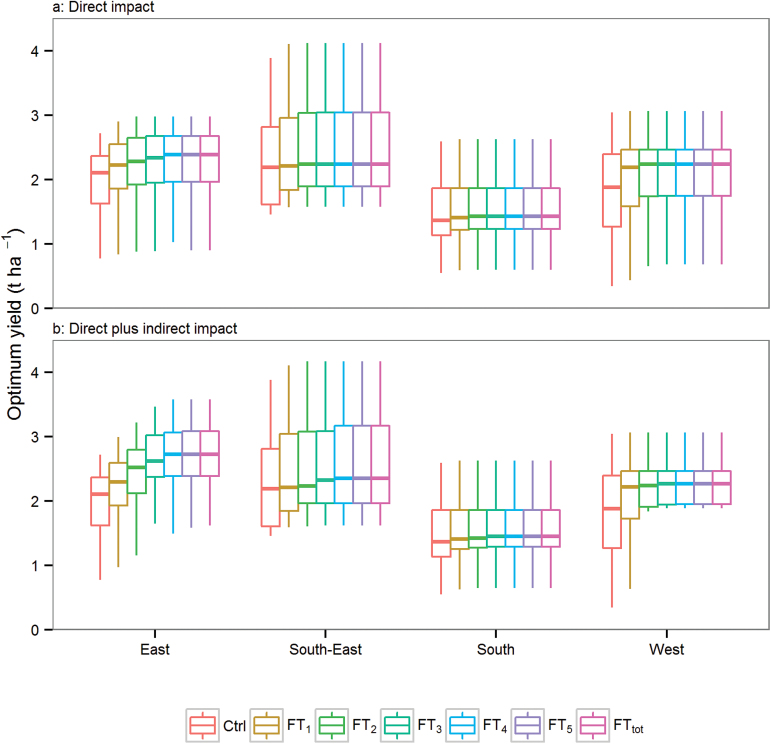
‘Direct’ (a) and ‘direct plus indirect’ (b) frost impact on yield in each region for mid-maturing cultivars with an estimated frost tolerance to 0 °C (Control), and for virtual genotypes of the same phenology with improved frost tolerance to –1 °C (FT_1_), –2 °C (FT_2_), –3 °C (FT_3_), –4 °C (FT_4_), –5 °C (FT_5_), or total frost tolerance (FT_tot_). Direct impact refers to yields of wheat crops sown at the optimum sowing time for current cultivars (Ctrl). Direct plus indirect impact refers to yield of crops sown at the optimum sowing time that is specific to each genotype with respect to its level of frost tolerance. Simulated yield for 60 locations (Fig. 1) from 1957 to 2013.

A highly sought alternative to reduce frost impact would be to develop varieties with increased levels of frost tolerance. Unfortunately, no useful improved post-heading frost tolerance has yet been discovered at least in part due to the practical difficulties in phenotyping large numbers of genotypes (Fredericks *et al*., 2012). With current cultivars considered sensitive to *T*_min_<0 °C, simulated average yields for the period from 1957 to 2013 ranged from 0.3 t ha^–1^ to 4.1 t ha^–1^ for sowing times related to the best long-term mean yield (Fig. 5; Supplementary Figs S7, S8 at *JXB* online). By totally removing the sensitivity of a genotype (FT_tot_) but retaining the same current sowing times (‘direct impact’), an average yield increase of 0.27, 0.21, 0.07, and 0.24 t ha^–1^ was simulated in the East, South-East, South, and West areas, respectively (Fig. 5a). An extra yield advantages of 0.52, 0.08, 0.06, and 0.11 t ha^–1^ was simulated on average in the East, South-East, South, and West areas, respectively, when adapting the sowing times (‘direct plus indirect impact’; Fig. 5b). These results highlight a strong interaction between genotype (frost tolerance) and management (sowing times), especially for the East (Fig. 5), indicating that gains due to the ability to advance sowing dates in the East are likely to be greater than the advantage from reduced frost damage *per se*.

**Fig. 6.**
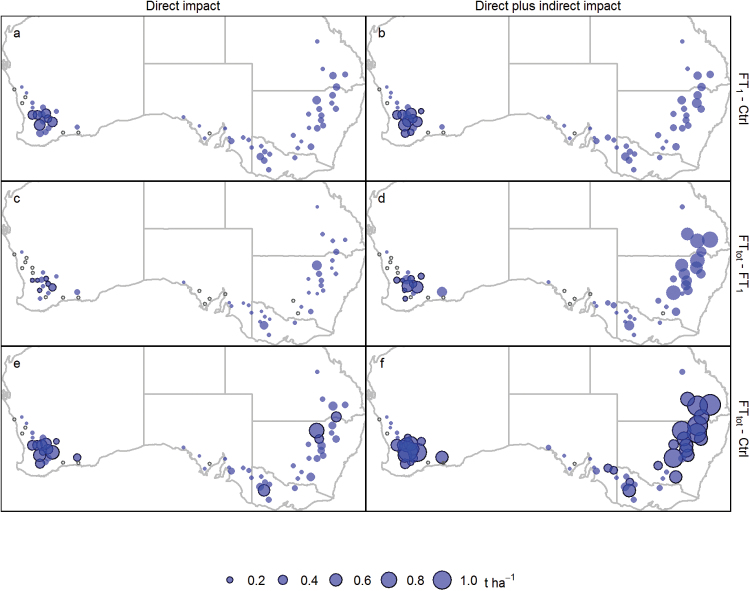
Simulated mean yield advantage of mid-maturing cultivars when (i) increasing the frost tolerance to –1 °C (i.e. FT_1_–Ctrl; a and b, top maps); (ii) considering the additional yield gain achieved with a total frost tolerance (i.e. FT_tot_ compared with –1 °C tolerance, i.e. FT_tot_–FT_1_; c and d, middle); and (iii) looking at the total yield advantage between total tolerance and the current level (FT_tot_–Ctrl; e and f, bottom). Simulations were done at 60 locations (Fig. 1) for sowing at the optimum sowing date for the current level of tolerance (0 °C, ‘control’; direct impact; a, c, and e, left) or at the optimum sowing date specific to each frost damage threshold level (direct plus indirect impact; b, d, and f, right). The size of the circle corresponds to the average yield increase (t ha^–1^) for 1957–2013. The open small circles indicate the sites where no yield advantage was simulated and black edges around blue circles indicate that yield advantage was significant (*P*<0.05).

The level of yield increase resulting from reduced frost sensitivities varied across the wheatbelt (Fig. 6). In the West, most of the potential simulated benefits were gained by reducing the frost damage threshold from 0 °C to just –1 °C with no change in management. In contrast, in the East and South-East, yield was substantially further improved by the frost tolerance to –3 °C or –4 °C, and extra yield improvement arose from the opportunity to exploit earlier sowing times and longer growing seasons (‘direct plus indirect impact’; Fig. 6). The greatest region-wide average yield impact was simulated in the East (0.79 t ha^–1^, representing a 38% increase) for frost tolerance to –4 °C and adjusted sowing date (Fig. 5b). Locally, even greater yield advantages were predicted for parts of the East and the West, with up to 1 t ha^–1^ yield increase (Fig. 6). Similar trends were observed for early- and late-maturing cultivars (Supplementary Figs S7–10).

### Small changes in the frost tolerance could increase national yield

**Fig. 7.**
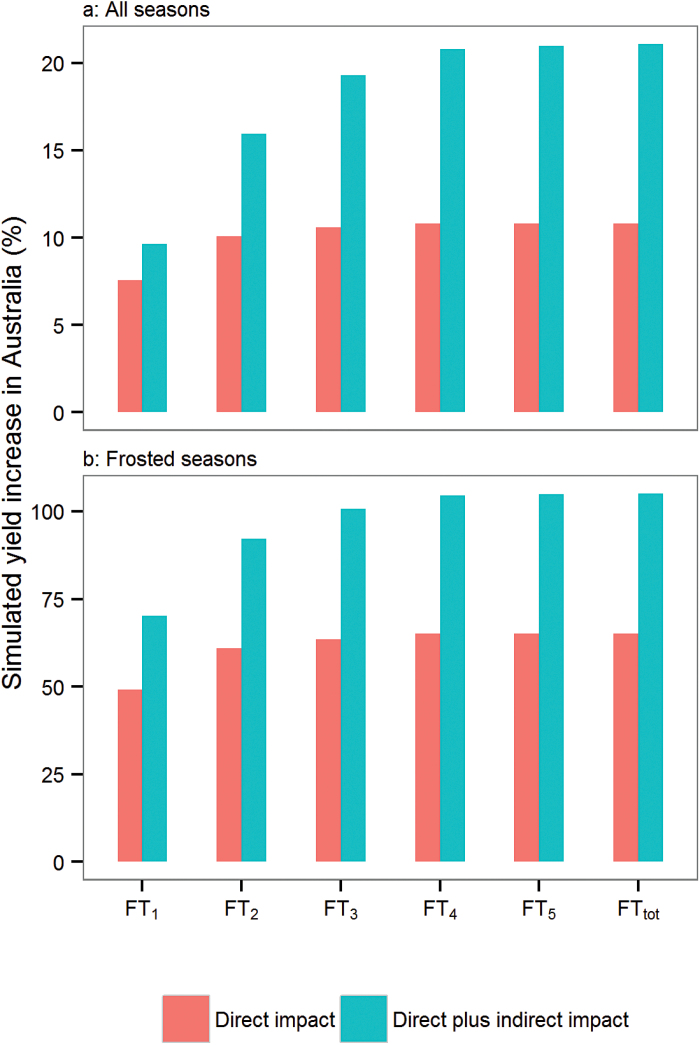
National yield advantage over all seasons (a) or only frosted seasons (b) of increased frost tolerance to –1 °C (FT_1_) to total frost tolerance (FT_tot_) for mid-maturing cultivars. Yield advantages were considered when changing either the frost-damage threshold temperature (direct impact; red) or both the frost-damage threshold temperature and the management (direct plus indirect effect; blue). Data correspond to the average over the 60 locations for simulations from 1957 to 2013 (37.3 million simulations in total; 10.1 million frosted simulated crops).

At the national scale, mean yield across 37.3 million simulations increased by 8.0% for a –1 °C frost tolerance (FT_1_) up to 10.8% for total frost tolerance (FT_tot_) for mid-maturing cultivars (direct impact; Fig. 7a) planted at the locally optimum sowing date. In practice, farmers sow their crops over a wider period, making frost tolerance worth 10.9% (FT_1_) to 18.5% (FT_tot_) of yield gain for mid-maturing cultivars with –1 °C to total frost tolerance, respectively (Supplementary Fig. S12 at *JXB* online). When the optimum sowing dates of the new genotypes were adjusted to reduce or avoid end-of-season stresses such as drought, yield increased by between 9.7% for –1 °C frost tolerance and 21.1% for total tolerance (direct plus indirect impact; Fig. 7a). Hence, adapting management practices (sowing times) resulted in an additional yield advantage of 1.7–10.3% for –1 °C and total tolerance, respectively (Fig. 7a). When only considering the crops (location×year×management×cultivar combinations) for which frost occurred during the sensitive period (11.1 million simulations, i.e. about one out of three crops), mean yield benefit from a –1 °C frost tolerance was >50% of the baseline yields for direct impact (FT_1_; Fig. 7b). Adapting the sowing date for those crops led to a nationwide benefit averaged across years of >100% from baseline yield when ≥3 °C tolerance was considered (FT_3_–FT_5_ and FT_tot_; Fig. 7b).

Nationwide, the greatest direct impact gain in yield (Fig. 7a) was achieved when decreasing the threshold temperature from 0 °C (Control) to –1 °C (FT_1_), while smaller further yield advantages were simulated by increasing the tolerance level to –2 °C (FT_2_) and –3 °C (FT_3_). Increasing frost tolerance to –4 °C and further resulted in little if any yield gain in terms of direct frost impact. In contrast, yield substantially increased up to a tolerance level of –4 °C when considering both direct and indirect gains (Fig. 7a). A similar trend in yield gains was estimated for all the tested genotypes regardless of their maturity type (Supplementary Fig. S11 at *JXB* online). Total frost tolerance increased the average simulated yield of early-, mid-, and late-maturing cultivars by as much as 9.8, 10.8, and 10.7% for direct impact, and 19.6, 21.1, and 18.2% for direct plus indirect impact (Fig. 7a; Supplementary Fig. S11). When considering the whole sowing window (without adjustment for the maturity type), national yield advantages were 25.3, 18.5, and 9.9% for early-, mid-, and late-maturing cultivars, respectively (Supplementary Fig. S12), highlighting that current genotypes sown early can be severely affected by frost in Australia, which is consistent with current observations.

## Discussion

### Occurrence of frost in the Australian wheatbelt

Since 1957, the number of frost events observed has significantly increased (*P*>0.1) in ~20% of the Australian wheatbelt mainly in the South-East, South, and West, and significantly decreased in about one-third of the wheatbelt, mainly in the East (Fig. 2). Other researches indicate an increase in minimum temperatures in Australia (Easterling *et al.*, 1997; Burrows *et al.*, 2011). Stone *et al.* (1996) reported significant reductions in the number of frost events from 1894 to 1992 in the East for temperatures from –3 °C to +3 °C, which were also observed for the shorter and more recent period from 1957 to 2013 in parts of this region, but more frost events were also found to be occurring in a few locations of the East, particularly for 0 °C and –1 °C (Fig. 2). While frost events might be related to altitude (Boer *et al.*, 1993), significant increases in frost events (0.35 d year^–1^) have also been observed in the South-East for elevations lower than 100 m (Crimp *et al.*, 2015). Other factors influencing the spatial and temporal patterns of minimum temperature in Australia include greenhouse gas emissions, solar radiation, the El Niño Southern Oscillation (ENSO), the position and intensity of subtropical highs, and blocking high pressure systems in Southeast Australia (Crimp *et al.*, 2015). For instance, frost events often occur during El Niño years, which are drier than average and may tend to become more frequent due to climate change (Stone *et al.*, 1996; Alexander and Hayman, 2008). However, last frost days are not related to ENSO (Alexander and Hayman, 2008).

### Simulated frost impacts increased over the last decades in major parts of the Australian wheatbelt

Over the last 57 years (1957–2013), simulated yield decreased by up to 1–2% year^–1^ in certain regions due to frost impacts. Frost impacts on wheat yield are related not only to temperature but also to phenology, particularly heading time. Significant trends in yield reduction due to higher frost impact were simulated in locations where no significant trend in last frost days was observed (Figs 2, 3). This was explained by a rate of last frost lagging behind phenological advance, which was hastened by increasing average temperature during winter at those locations (data not shown). Unless genotype maturities are modified, global warming may result in crops reaching the highly susceptible post-heading stage more rapidly, and thus becoming vulnerable to greater yield loss (Gu *et al.*, 2008; Zheng *et al.*, 2012).

### Limitation of crop modelling broad-scale simulations of frost impact

Post-heading frost damage is rarely accurately recorded at the farm level given the difficulty of estimating yields that would have been achieved in the absence of frost. It is even more difficult to estimate losses at the shire levels due to the high variation in radiant frosts with local topography (Dixit and Chen, 2010, 2011). In the absence of accurate empirical records, it is believed that the current study provides a useful estimate of the extent and economic impact of frosts for wheat in Australia. However, some caution is needed in interpreting some aspects of the simulations. First, it is important to note that the current study is based on current expert opinion and uses a yield multiplier of 0.1 for each frost event during the sensitive heading period, which may overestimate the actual impacts of frosts, particularly for mild frost events at or around 0 °C. Secondly, this study uses historical weather data with minimum temperatures recorded in Stevenson screens. Temperatures measured in this way have been found to be an imperfect measure of canopy temperature and crop damage (Hayman *et al.*, 2007; Frederiks *et al.*, 2011). Stevenson screen data provide a good measure of bulk air temperature in most conditions but not during radiant frosts, as the temperature sensor is sheltered from radiant heat loss that affects the crop. Hence, during radiant frosts, canopy temperatures may drop well below temperatures recorded in the Stevenson screen, and damaging canopy temperatures can be reached when recorded ambient temperatures are near zero. However, when there is air movement or partial cloud cover, the canopy temperature and Stevenson screen temperatures may both be near zero but frost damage to the crop is unlikely (Frederiks *et al.*, 2012). Thirdly, radiant frosts can vary greatly within a single farm depending on the topography (Dixit and Chen, 2010, 2011). Overall, the baseline temperature of 0 °C used in this study is conservative and may overestimate the occurrence and yield impact of damaging frosts in certain conditions. For this reason, data were presented for a range of frost intensities, making it is possible to determine the incidence of more severe frost events and the yield effect related to lower baseline temperatures, if required. For instance, the virtual genotype with improved frost tolerance to –1 °C (FT_1_) can be used as a proxy for a revised baseline temperature of –1 °C in conditions where Stevenson screen temperatures are closer to canopy temperatures than assumed in this broad-scale study.

### Managing frost risks

With limited practical management options, farmers reduce frost damage by adopting relevant combinations of planting dates and cultivars to minimize post-heading frost risks (Woodruff, 1992; van Rees *et al.*, 2014). However, in Australia, postponing heading typically increases risks of drought and heat stress during grain filling, and thus reduces yield potential (Asseng *et al.*, 2011; Chenu *et al.*, 2011, 2013). In the northern part of the wheatbelt, dramatic increases in yield have been observed when mid-winter flowering wheat crops escape frost (Woodruff and Tonks, 1983; Frederiks *et al.*, 2011). Improving frost tolerance and advancing sowing times can greatly benefit yield in regions like the eastern wheatbelt. The simulations suggest mean yield increases of >1 t ha^–1^ are possible at some locations in the eastern wheatbelt for fully tolerant genotypes (FT_tot_) planted early when compared with current cultivars planted at their current optimal sowing times (Fig. 6f).

With global warming, crop growing seasons are expected to shorten and will potentially increase yield loss due to frost if the last frost day advances more slowly than the heading date. The present observations suggest that such a trend has occurred over recent decades in parts of the wheatbelt (Fig. 4). In the future, longer season varieties could be required to maintain the current crop-growing duration (Zheng *et al.*, 2012). Gene-based models can assist in exploring the possible combinations of known phenology genes and to identify promising genotypes for target environments (White *et al.*, 2008; Bertin *et al.*, 2010; Zheng *et al.*, 2013). Combined with future climatic predictions, such modelling can help predict the impacts of climate change and minimize frost impacts to levels acceptable to farmers (Chapman *et al.*, 2012; Zheng *et al.*, 2012).

### Breeding for frost tolerance

Given variations recorded in minimum temperatures (Figs 2, 3; Supplementary Fig. S2 at *JXB* online) and frost impacts (Fig. 4; Supplementary Figs S4, S5), different levels of tolerance seem required to best benefit producers in the East versus the West of Australia (Fig. 6). In the West, most of the simulated yield advantage was achieved by reducing the damage threshold temperature from 0 °C to –1 °C without the need to adapt sowing dates (Fig. 6). In the East, a substantial yield increase was similarly simulated for improving frost tolerance from 0 °C to –1 °C, but also from –1 °C to –2 °C, and from –2 °C to –3 °C. In addition, improved genetic frost tolerance would allow earlier sowing and result in extra yield gain, especially in the East. As previously mentioned, temperatures used in this study were recorded in the Stevenson screen, and are thus imperfect to estimate canopy temperature and crop damage. It is worthwhile to note that the current study may overestimate damage, particularly for temperatures close to zero, which is particularly important when interpreting the results for the West where a large effect was predicted for a change in the damage threshold from 0 °C to –1 °C.

While vegetative-frost tolerance is relatively well understood in wheat (e.g. Ganeva *et al.*, 2013; Pearce *et al.*, 2013; Zhao *et al.*, 2013; Case *et al.*, 2014; Zhu *et al.*, 2014), understanding of post-heading frost is less advanced. In barley, quantitative trait loci (QTLs) for post-heading frost tolerance were identified in chromosomes 2H and 5H (Reinheimer *et al.*, 2004; Chen *et al.*, 2009a, b). However, Frederiks *et al.* (2011) demonstrated that these alleles are not likely to provide useful sources of frost tolerance or markers to develop improved varieties.

Breeding for post-heading frost tolerance is limited partly because of the difficulties in phenotyping multiple genotypes in similar conditions (Frederiks *et al.*, 2012). Variations in frost damage within a trial can be caused by small variations in phenology, or small variations in minimum temperature (Frederiks *et al.*, 2012). Thus, breeders are obliged to select for frost tolerance in dedicated trials. A field-based phenotyping method was recently developed using artificial lights to impose a photoperiod extension gradient that brings genotypes of different phenology to a common developmental stage and allows testing in naturally occurring spring radiant frosts (Frederiks *et al.*, 2012). By allowing frost damage to be scored for varieties at the same stage, it is hoped that this phenotyping method can identify types with improved post-heading tolerance. Originally developed to characterize frost tolerance for small numbers of lines in physiological trials, the method is currently being adapted to increase throughput and allow phenotyping of larger numbers of lines.

### Conclusion

Catastrophic yield losses caused by post-heading frosts have been reported by wheat producers in Australia and many other cropping regions of the world. The crop model APSIM was adapted and used to estimate the frost impact on wheat yield in Australia. In the absence of accurate empirical records of the extent of losses caused by frosts in Australia, it is believed that the current study provides valuable insights into the impact of frosts. Overall, the results explained the major recent incidences of frost damage in wheat fields based on expert knowledge and gave results consistent with variety planting recommendations.

Simulated results suggest that the frequency of frost events increased and last frost days occurred later in major areas of the Australian wheatbelt between 1957 and 2013. In addition, in part of the wheatbelt, an increase in average temperatures resulted in a significant increase in frost impact on yield due to the more rapid advance in development of crops to sensitive stages than the advance in last frost date. These changes over the last six decades suggest that, far from alleviating the problem of frost due to higher average night-time temperatures, climate changes may be increasing frost in important cropping areas.

This simulation study suggests that national yield advantage of up to 20% could result from the breeding of frost-tolerant lines if useful genetic variation can be found. In the simulations, reducing frost sensitivity by 1 °C resulted in major yield improvement across the Australian wheatbelt while additional advantages could be achieved in the East for frost tolerance to as low as –3 °C or –4 °C. In addition, substantial further gains from frost-tolerant lines arose from earlier crop sowing, especially in the East.

Thus, trends in climate over the past six decades do not indicate that frost risk is decreasing. Breeding for post-heading frost tolerance should remain a high priority for the Australian wheat industry despite global warming.

## Supplementary data

Supplementary data are available at *JXB* online

Figure S1. Number of annual frost days (*T*_min_<0 °C) against last frost days (90th percentile) for each region of the wheatbelt. Data for 1957–2013, for each of the points of the 0.05° gridded weather data set.

Figure S2. The spatial distribution of the mean and temporal trend for minimum temperature across the Australian wheatbelt from 1957 to 2013 (from May to October).

Figure S3. Maps of simulated yield reduction, frequency of years when yield reduction is >10% of years, and temporal trends of yield reduction caused by frost events (*T*_min_< –1 °C) for the mid-maturity cultivar Janz.

Figure S4. Maps of the yield reduction, frequency of years when yield reduction is >10% of years, and temporal trends of yield reduction caused by frost events (*T*_min_<0 °C) for the early-maturity cultivar Axe.

Figure S5. Maps of the yield reduction, the frequency of years when yield reduction is >10% of years, and temporal trends of yield reduction caused by frost events (*T*_min_<0 °C) for the late-maturity cultivar Sunbrir.

Figure S6. Impact of sowing date and cultivar on the timing of flowering compared with the occurrence of extreme temperature events.

Figure S7. ‘Direct’ and ‘direct plus indirect’ frost impacts on yield in each region for an early-maturing cultivar.

Figure S8. Direct and direct plus indirect frost impact on yield in each region for a late-maturing cultivar.

Figure S9. Simulated yield advantage of an early-maturing cultivar when (i) reducing frost damage threshold temperatures to –1 °C; (ii) considering the additional yield gain achieved with a total frost tolerance; and (iii) looking at the total yield advantage between total tolerance and the current level.

Figure S10. Simulated yield advantage of a late-maturing cultivar when (i) reducing frost damage threshold temperatures to –1 °C; (ii) considering the additional yield gain achieved with a total frost tolerance; and (iii) looking at the total yield advantage between total tolerance and the current level.

Figure S11. National yield advantage of reduced damage threshold temperatures of –1 °C (FT_1_) to total frost tolerance (FT_tot_) for an early- and late-maturing cultivar.

Figure S12. National ratio of yield advantage of reduced damage threshold temperatures of –1 °C (FT_1_) to total frost tolerance (FT_tot_) for an early-, mid-, and late-maturing cultivar.

Supplementary Data
