# Supplementary material for: Frost trends and their estimated impact on yield in the Australian wheatbelt
Source: J Exp Bot. 2015 Apr 28;66(12):3611–23. doi: 10.1093/jxb/erv163 (PMC4463805; doi:10.1093/jxb/erv163)
Supplement: Supplementary Data [file supp_66_12_3611__index.html]

Frost trends and their estimated impact on yield in the Australian wheatbelt — Frost trends and their estimated impact on yield in the Australian wheatbelt — Supplementary Data 

# Frost trends and their estimated impact on yield in the Australian wheatbelt

## Supplementary Data

Data files

**Files in this Data Supplement:**

- Supplementary Data - Supplementary Data
