# Supplementary material for: Frost trends and their estimated impact on yield in the Australian wheatbelt
Source: J Exp Bot. 2015 Apr 28;66(12):3611–23. doi: 10.1093/jxb/erv163 (PMC4463805; doi:10.1093/jxb/erv163)
Supplement: Supplementary Data [file supp_erv163_jexbot141812_file001.pdf]

Frost trends and their estimated impact on yield in the Australian wheatbelt

Bangyou Zheng[1], Scott C Chapman[1], Jack T Christopher[2], Troy M Frederiks[3], Karine Chenu[4*]

[1] CSIRO Agriculture Flagship, Queensland Bioscience Precinct, 306 Carmody Road, St. Lucia, QLD 4067, Australia

[2] The University of Queensland, Queensland Alliance for Agriculture and Food Innovation (QAAFI) , Leslie Research Facility, PO Box 2282 Toowoomba, QLD, 4350, Australia

[3] Queensland Department of Agriculture, Fisheries and Forestry (DAFFQ), Leslie Research Facility, PO Box 2282 Toowoomba, QLD, 4350, Australia

[4] The University of Queensland, Queensland Alliance for Agriculture and Food Innovation (QAAFI), 203 Tor Street, Toowoomba, QLD 4350, Australia

[*] Corresponding Author: Karine Chenu, Tel: +61 (0)7 4688 1357, email: karine.chenu@uq.edu.au

Table S1 Locations and soils chosen to represent the Australian wheatbelt including sowing windows and optimum sowing times. The optimum sowing times represent for medium cultivars under the control scenarios (Ctrl, i.e. without frost tolerance). Information concerning other soil characteristics, initial soil water content and fertilisation levels used in the simulations are available in Chenu et al. (2013).

Region	State	Site	Latitude	Longitude	Soil classification	Sowing window		Optimum sowing time		
						Start	End	Early	Medium	Late
East	QLD	Goondiwindi	-28.55	150.31	Red brown	1-May	21-Jun	22-May	16-May	04-May
		Meandarra	-27.32	149.88	Grey vertosol	1-May	21-Jun	01-Jun	26-May	10-May
		Dalby	-27.18	151.26	Grey vertosol	1-May	21-Jun	07-Jun	03-Jun	27-May
		Roma	-26.57	148.79	Brown vertosol	1-May	21-Jun	04-Jun	30-May	13-May
		Emerald	-23.53	148.16	Black vertosol	15-Apr	7-Jun	17-May	12-May	27-Apr
	NSW	Condobolin	-33.07	147.23	Sandy loam	1-May	21-Jun	20-Jun	18-Jun	23-May
		Wellington	-32.80	148.80	Sandy clay loam	1-May	21-Jun	20-Jun	03-Jun	09-May
		Dubbo	-32.24	148.61	Red dermosol	1-May	21-Jun	19-Jun	02-Jun	12-May
		Gilgandra	-31.71	148.66	Brown dermosol	1-May	21-Jun	10-Jun	26-May	09-May
		Nyngan	-31.55	147.20	Sandy clay loam	1-May	21-Jun	21-May	18-May	02-May
		Coonamble	-30.98	148.38	Sandy clay	1-May	21-Jun	29-May	17-May	07-May
		Gunnedah	-30.98	150.25	Black vertosol	1-May	21-Jun	17-Jun	04-Jun	13-May
		Narrabri	-30.32	149.78	Grey vertosol	1-May	21-Jun	20-Jun	12-Jun	17-May
		Walgett	-30.04	148.12	Grey vertosol	1-May	21-Jun	26-May	10-May	01-May
		Moree	-29.48	149.84	Grey vertosol	1-May	21-Jun	04-Jun	29-May	17-May
South-East		Urana	-35.33	146.03	Clay loam	1-May	21-Jun	17-Jun	27-May	17-May
		WaggaWagga	-35.16	147.46	Red sodosol	1-May	21-Jun	21-Jun	17-Jun	07-Jun
		Yanco	-34.61	146.42	Brown sodosol	1-May	21-Jun	15-Jun	31-May	23-May
		Merriwagga	-33.92	145.52	Sandy loam	1-May	21-Jun	04-Jun	23-May	09-May
		Parkes	-33.14	148.16	Sandy clay loam	1-May	21-Jun	21-Jun	03-Jun	30-May
	VIC	LakeBolac	-37.71	142.84	Sandy clay loam	1-May	21-Jun	21-Jun	21-Jun	21-Jun
		Longerenong	-36.67	142.30	Clay	1-May	21-Jun	21-Jun	06-Jun	22-May
		Dookie	-36.37	145.70	Loam	1-May	21-Jun	21-Jun	03-Jun	19-May
		Glenlee	-36.26	141.86	Clay	1-May	21-Jun	20-Jun	07-Jun	23-May
		Birchip	-35.98	142.92	Clay loam	1-May	21-Jun	09-Jun	24-May	12-May
		Hopetoun	-35.73	142.37	Loamy sand	1-May	21-Jun	14-Jun	26-May	08-May
South		Walpeup	-35.12	142.00	Loamy sand	1-May	21-Jun	03-Jun	22-May	14-May
	SA	Pinnaroo	-35.26	140.91	Loamy sand	1-May	21-Jun	01-Jun	15-May	13-May
		Roseworthy	-34.53	138.69	Dark brown cracking clay	1-May	21-Jun	21-Jun	31-May	12-May
		Loxton	-34.44	140.60	Loamy sand	1-May	21-Jun	09-Jun	28-May	14-May
		Cummins	-34.26	135.73	Dark loamy clay	1-May	21-Jun	21-Jun	02-Jun	22-May
		Waikerie	-34.18	139.98	Hypercalcic calcarosol	1-May	21-Jun	28-May	22-May	08-May

Continued on next page

Region	State	Site	Latitude	Longitude	Soil classification	Sowing window		Optimum sowing time		
						Start	End	Early	Medium	Late
		Balaklava	-34.14	138.42	Sandy loam	1-May	21-Jun	04-Jun	13-May	01-May
		Rudall	-33.69	136.27	Grey calcareous loamy sand	1-May	21-Jun	08-Jun	20-May	10-May
		PortPirie	-33.17	138.01	Loamy sand	1-May	21-Jun	05-Jun	22-May	22-May
		Minnipa	-32.84	135.15	Red light sandy clay loam	1-May	21-Jun	23-May	15-May	01-May
		Ceduna	-31.90	133.42	Grey calcareous sandy loam	1-May	21-Jun	23-May	16-May	09-May
West	WA	Katanning	-33.69	117.56	Deep sandy duplex	1-May	21-Jun	01-Jun	14-May	07-May
		Esperance	-33.60	121.78	Deep sandy duplex	1-May	21-Jun	27-May	14-May	06-May
		Ravensthorpe	-33.58	120.05	Deep sandy duplex	1-May	21-Jun	27-May	17-May	11-May
		Nyabing	-33.54	118.15	Clay	1-May	21-Jun	20-May	13-May	03-May
		LakeGrace	-33.10	118.46	Shallow sandy duplex	1-May	21-Jun	14-May	12-May	06-May
		SalmonGums	-32.99	121.62	Shallow sandy duplex	1-May	21-Jun	13-Jun	12-Jun	07-Jun
		Wickepin	-32.78	117.50	Gravel	1-May	21-Jun	13-May	05-May	02-May
		Hyden	-32.44	118.90	Deep sandy duplex	1-May	21-Jun	31-May	19-May	18-May
		Corrigin	-32.33	117.87	Deep sandy duplex	1-May	21-Jun	11-Jun	05-Jun	01-Jun
		Narembeen	-32.07	118.40	Loamy earth	1-May	21-Jun	26-May	20-May	12-May
		Cunderdin	-31.66	117.25	Deep sandy duplex	1-May	21-Jun	13-May	06-May	01-May
		Northam	-31.65	116.66	Loamy earth	1-May	21-Jun	02-Jun	22-May	15-May
		Kellerberrin	-31.62	117.72	Sandy earth	1-May	21-Jun	26-May	20-May	15-May
		Merredin	-31.50	118.22	Shallow loamy duplex	1-May	21-Jun	25-May	20-May	15-May
		SouthernCross	-31.23	119.33	Gravel	1-May	21-Jun	26-May	20-May	15-May
		WonganHills	-30.84	116.73	Deep loamy duplex	1-May	21-Jun	13-May	07-May	04-May
		Bencubbin	-30.81	117.86	Sandy earth	1-May	21-Jun	13-May	06-May	04-May
		Badgingarra	-30.34	115.54	Gravel	1-May	21-Jun	17-May	02-May	01-May
		Dalwallinu	-30.28	116.66	Sand	1-May	21-Jun	09-May	08-May	03-May
		Carnamah	-29.69	115.89	Sand	1-May	21-Jun	11-May	09-May	04-May
		Morawa	-29.21	116.01	Deep loamy duplex	1-May	21-Jun	15-May	10-May	05-May
		Geraldton	-28.80	114.70	Sand	1-May	21-Jun	08-May	06-May	08-May
		Mullewa	-28.54	115.51	Sandy loam	1-May	21-Jun	02-May	01-May	01-May

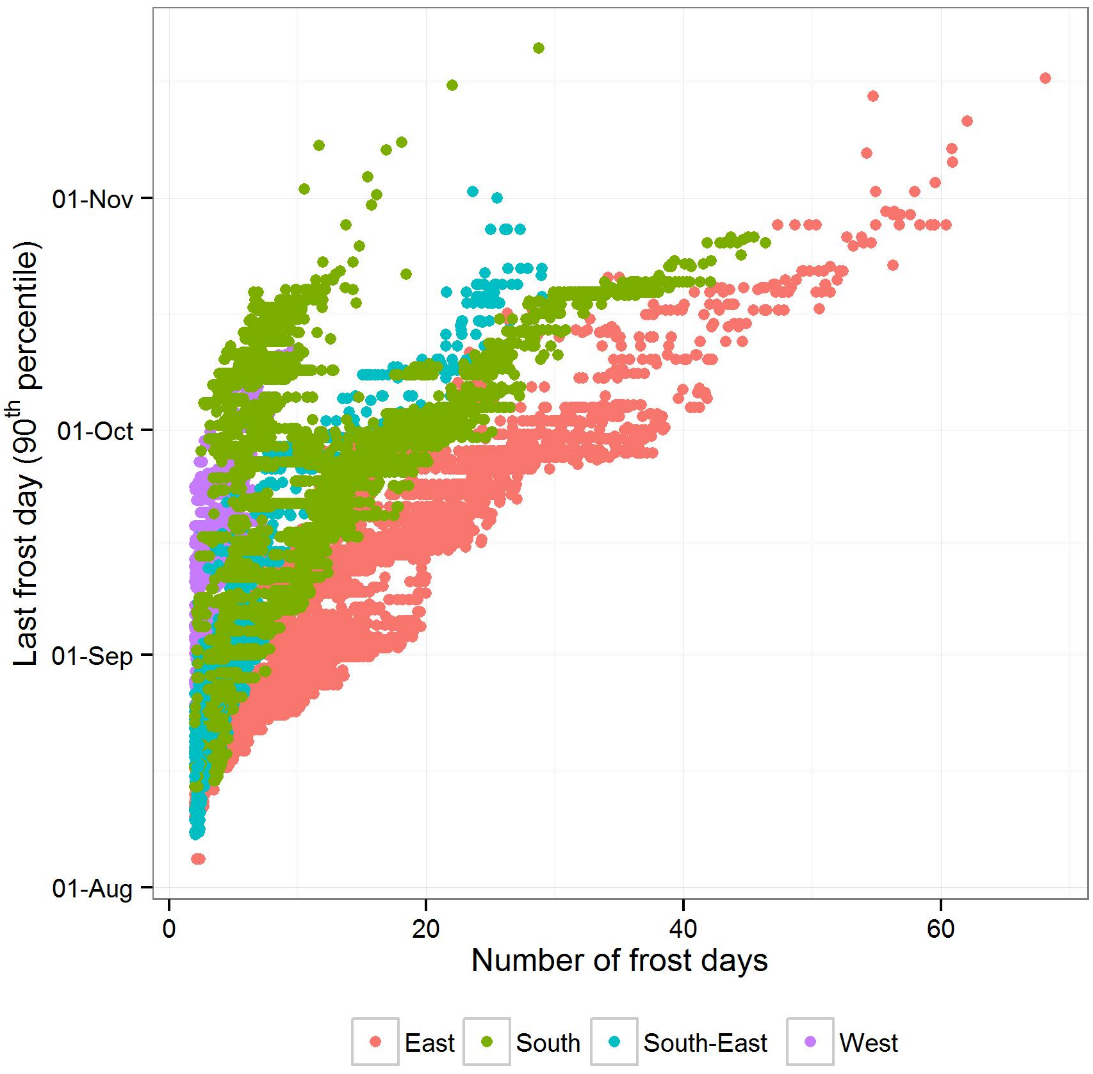

Fig. S1 Number of annual frost days ($T_{min} < 0°C$) against last frost days (90th percentile) for each region of the wheatbelt. Data for 1957-2013, for each of the points of the 0.05° gridded weather dataset.

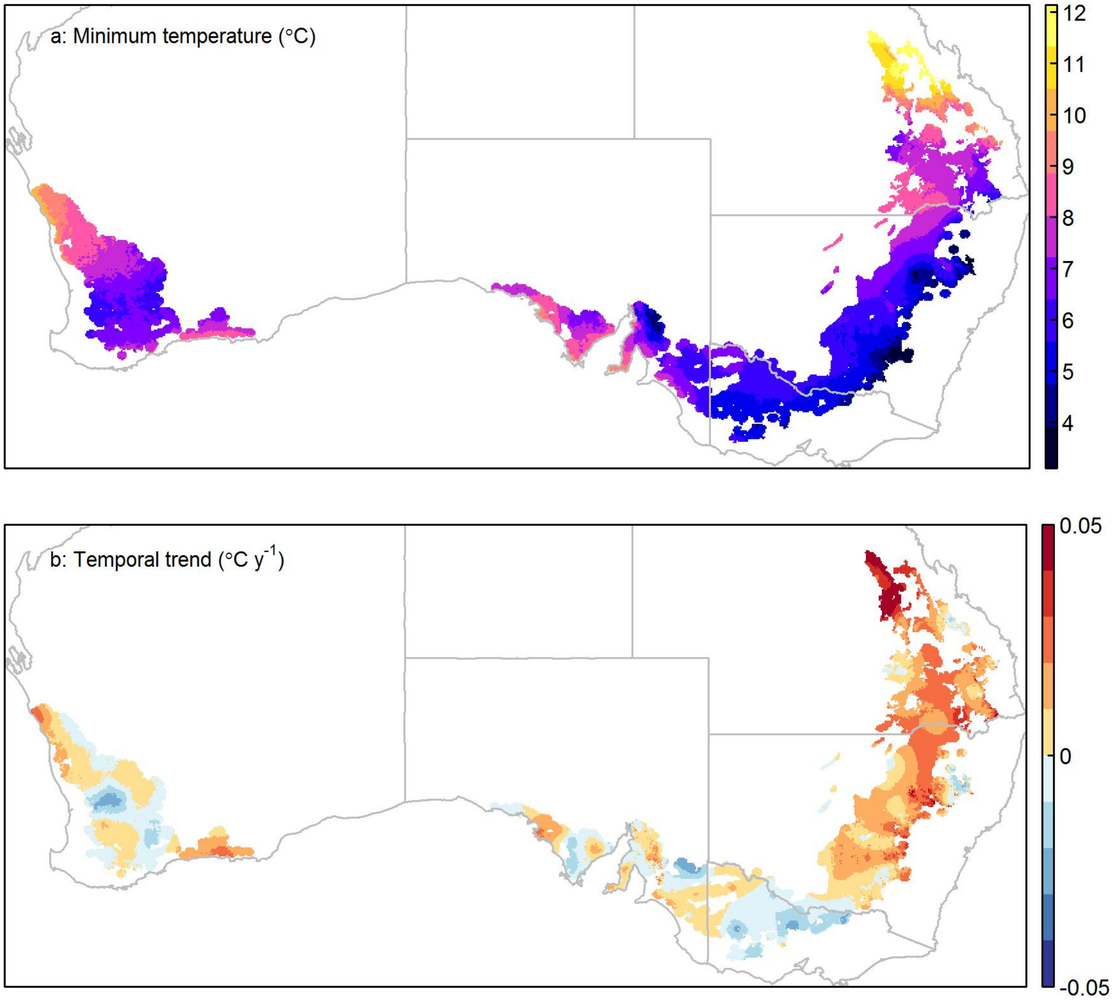

Fig. S2 The spatial distribution of mean (a), and temporal trend (b) for minimum temperature across the Australian wheatbelt from 1957 to 2013 (from May to October). Negative temporal trends correspond to a decrease in temperature.

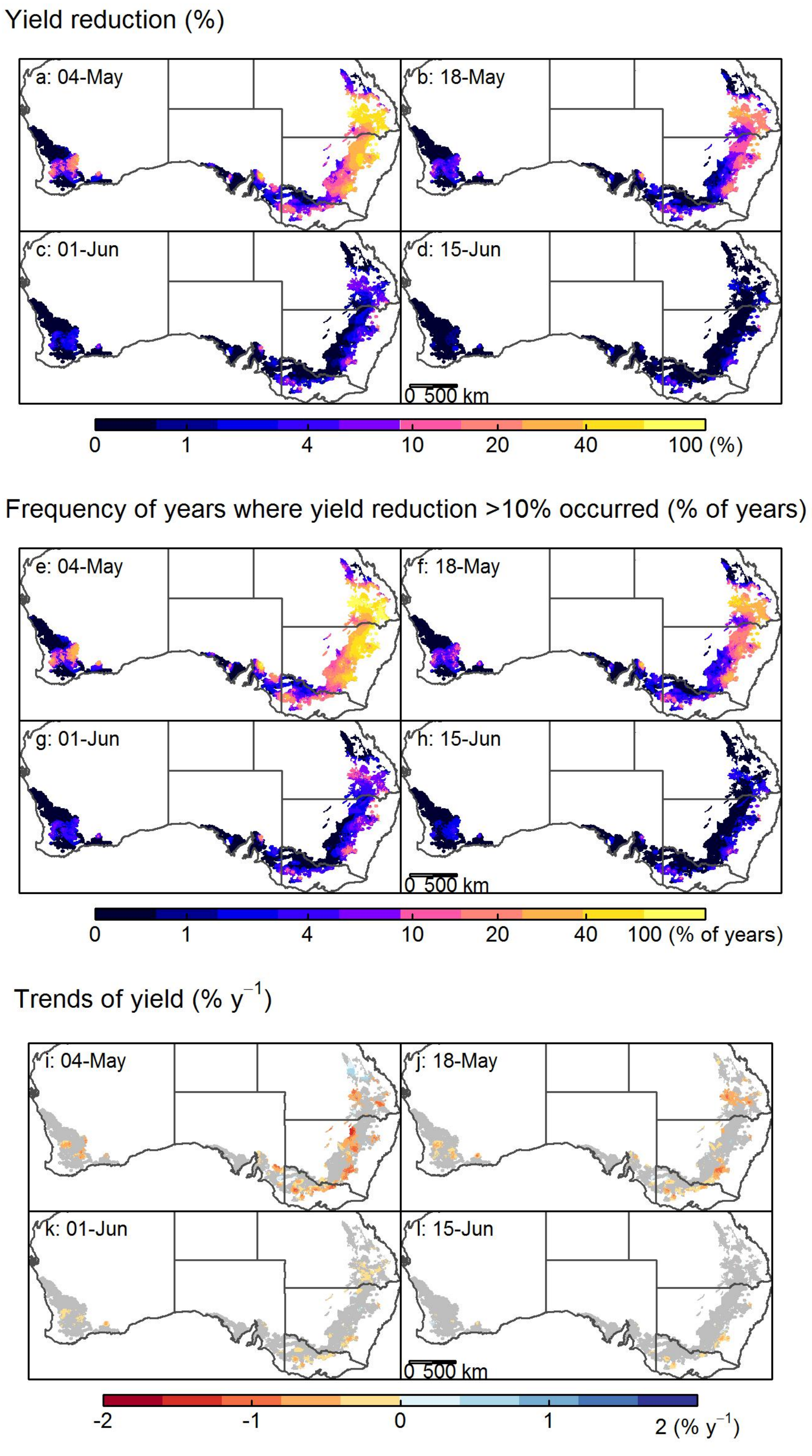

Fig. S3 Maps for the **mid-maturity cultivar Janz** of average simulated yield reduction due to frost (T_{min} < **-1°C**) in the 57 years from 1957 to 2013 (a-d), the frequency of years (% of years) when yield reduction greater than 10% occurred (e-h), and trends over time in yield (i-l) for sowing at 04-May (a, e and i), 18-May (b, f and j), 01-Jun (c, g and k) and 15-Jun (d, h and i) across the Australian wheatbelt.

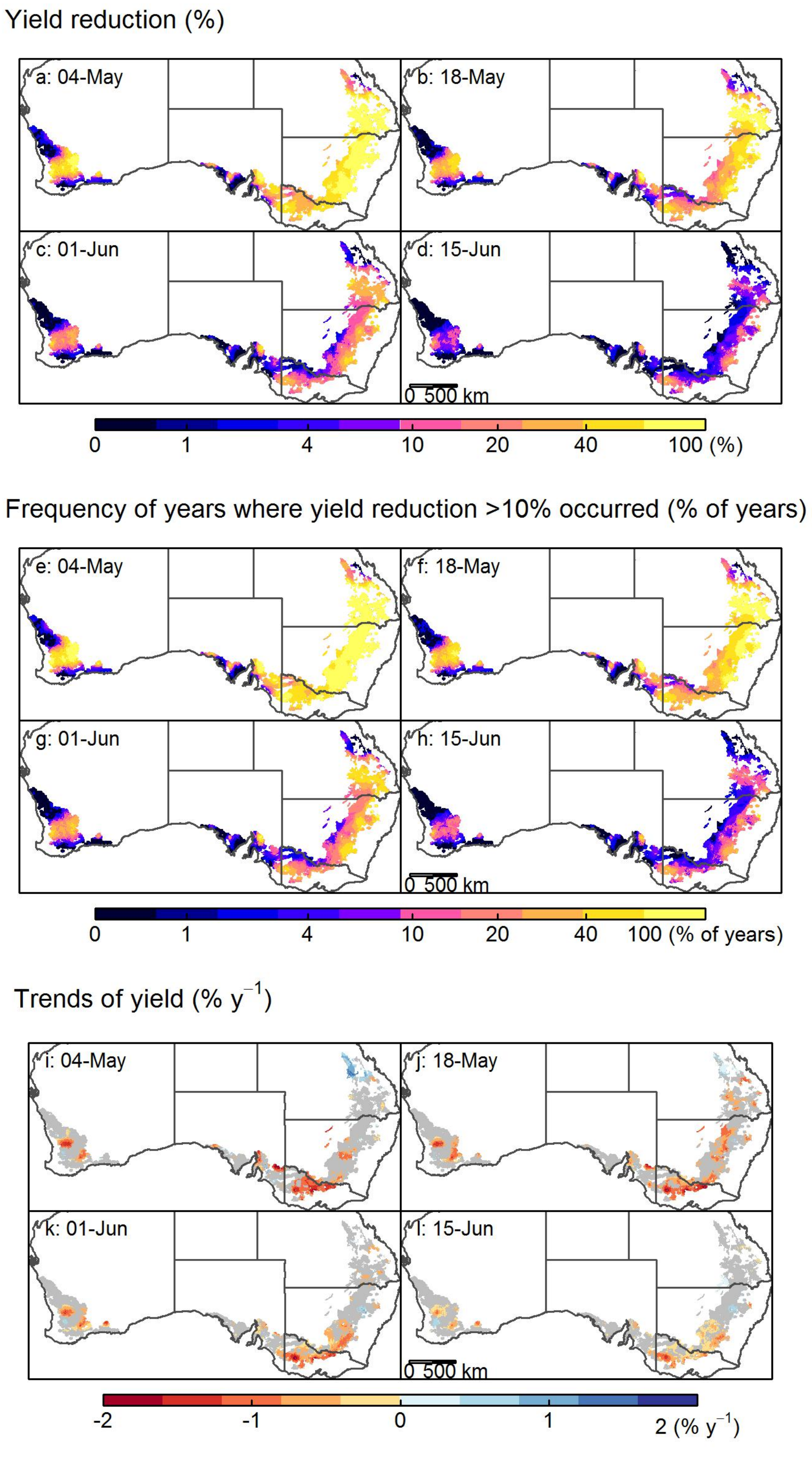

Fig. S4 Maps for the **short-maturity cultivar Axe** of average simulated yield reduction due to frost (T_{min} < **0°C**) in the 57 years from 1957 to 2013 (a-d), the frequency of years (% of years) when yield reduction greater than 10% occurred (e-h), and trends over time in yield (i-l) for sowing at 04-May (a, e and i), 18-May (b, f and j), 01-Jun (c, g and k) and 15-Jun (d, h and i) across the Australian wheatbelt.

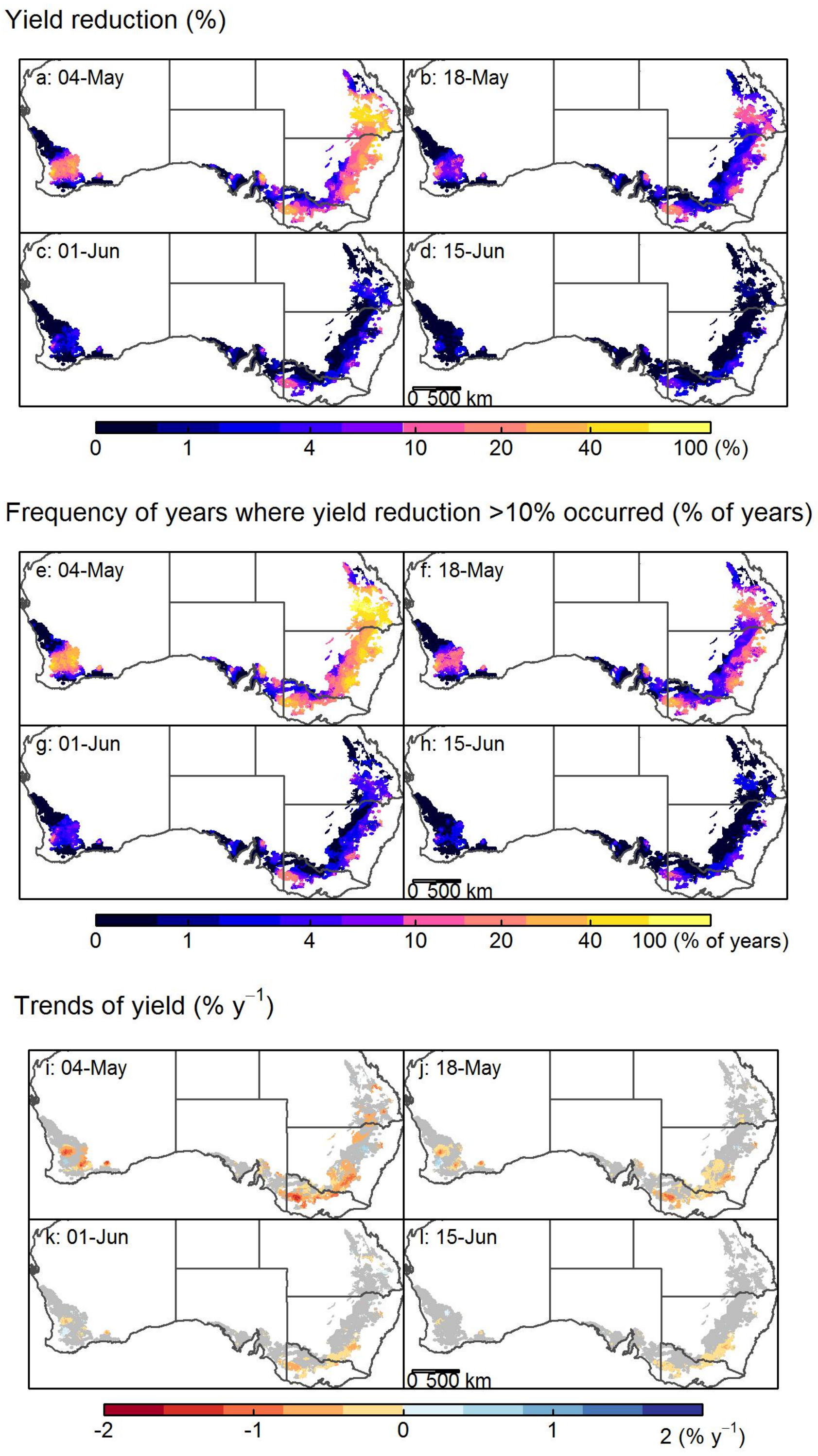

Fig. S5 Maps for the **late-maturity cultivar Sunbri** of average simulated yield reduction due to frost (T_{min} < **0°C**) in the 57 years from 1957 to 2013 (a-d), the frequency of years (% of years) when yield reduction greater than 10% occurred (e-h), and trends over time in yield (i-l) for sowing at 04-May (a, e and i), 18-May (b, f and j), 01-Jun (c, g and k) and 15-Jun (d, h and i) across the Australian wheatbelt.

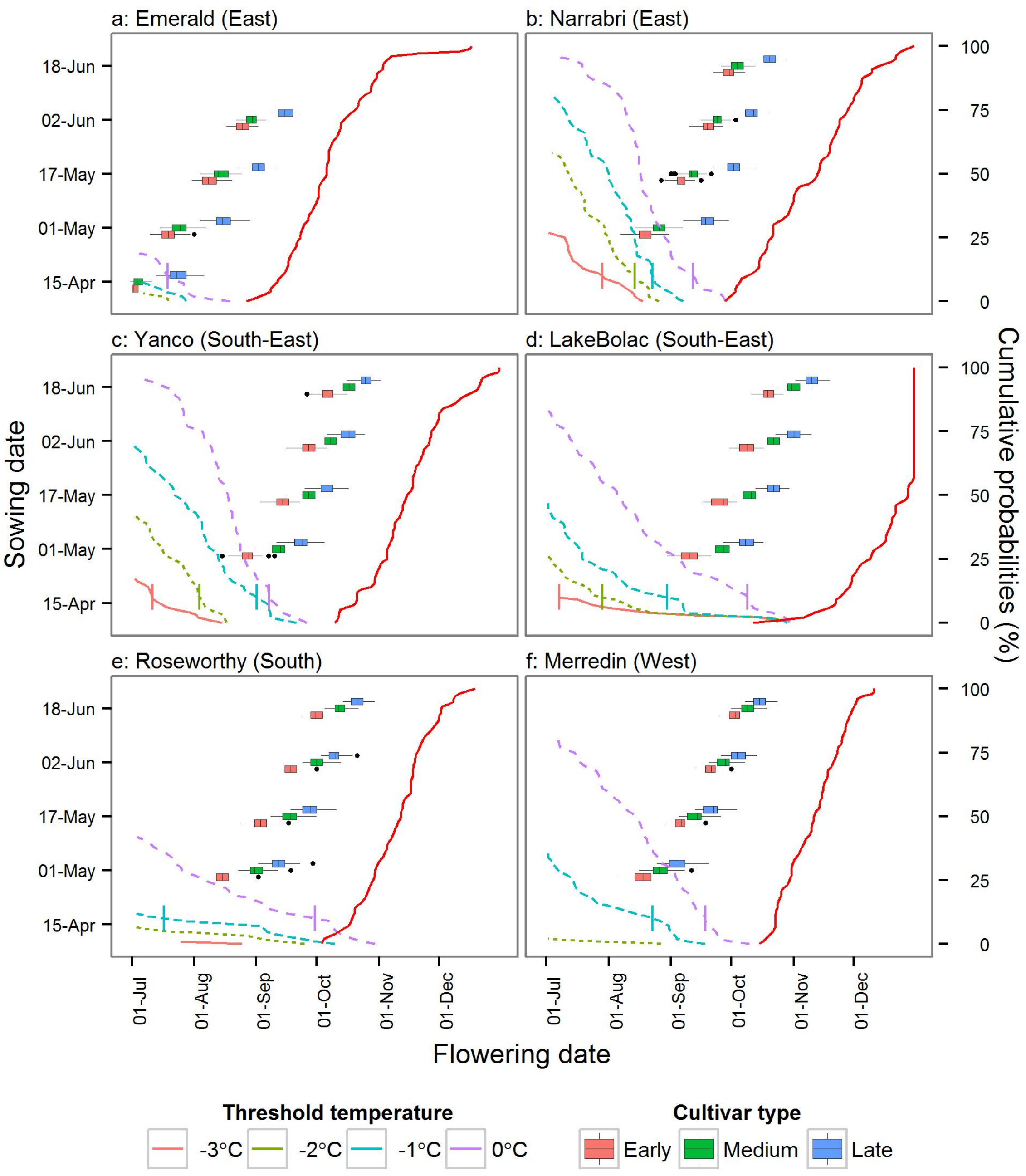

Fig. S6 Effect of sowing date and cultivar on the timing of flowering compared to the occurrence of extreme-temperature events (example for 6 key stations locations for 1957-2013. The boxplot shows the variation in flowering date (x-axis) for different sowing dates (y-axis: every two weeks from 20th April to 15th June) and for cultivars of different maturity (Early, Medium and Late, Table 1). The red solid line represents the probability distribution of first heat days from 1957 to 2013, which defined as the first day after winter with a maximum air temperature greater than 35°C . The colour dash lines correspond to the probability distribution of last frost days from 1957 to 2013, which defined as the last days of year with a minimum air

temperature below the specific threshold (-4, -2 and 0°C).

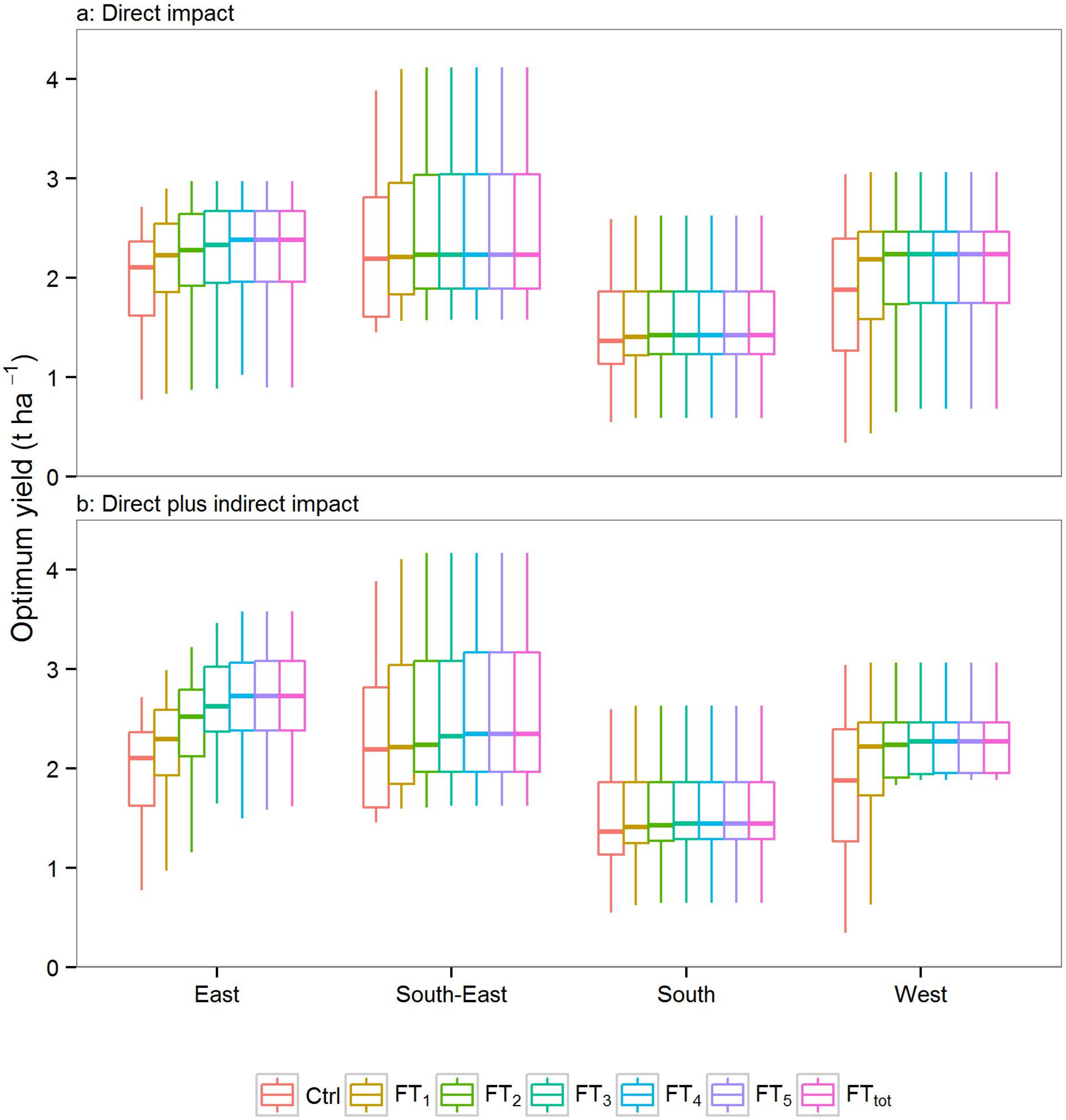

Fig. S7 Direct (a) and direct plus indirect (b) frost impact on yield in each region for a **early-maturing** cultivar with an estimated frost tolerance to 0°C (Control), and for virtual genotypes of the same phenology with improved frost tolerance to -1°C (FT_1), -2°C (FT_2), -3°C (FT_3), -4°C (FT_4), -5°C (FT_5) or total frost tolerance (FT_{tot}). Direct impact refers to yield gain of wheat with improved tolerance, which were sown at the optimum sowing time of current cultivars (Ctrl). Direct plus indirect impact refers to yield gain improved-tolerance crops sown at the optimum sowing time that is specific to each genotype with respect to its levels of frost tolerance. Simulated yield for 60 locations (Fig. 1) from 1957 to 2013.

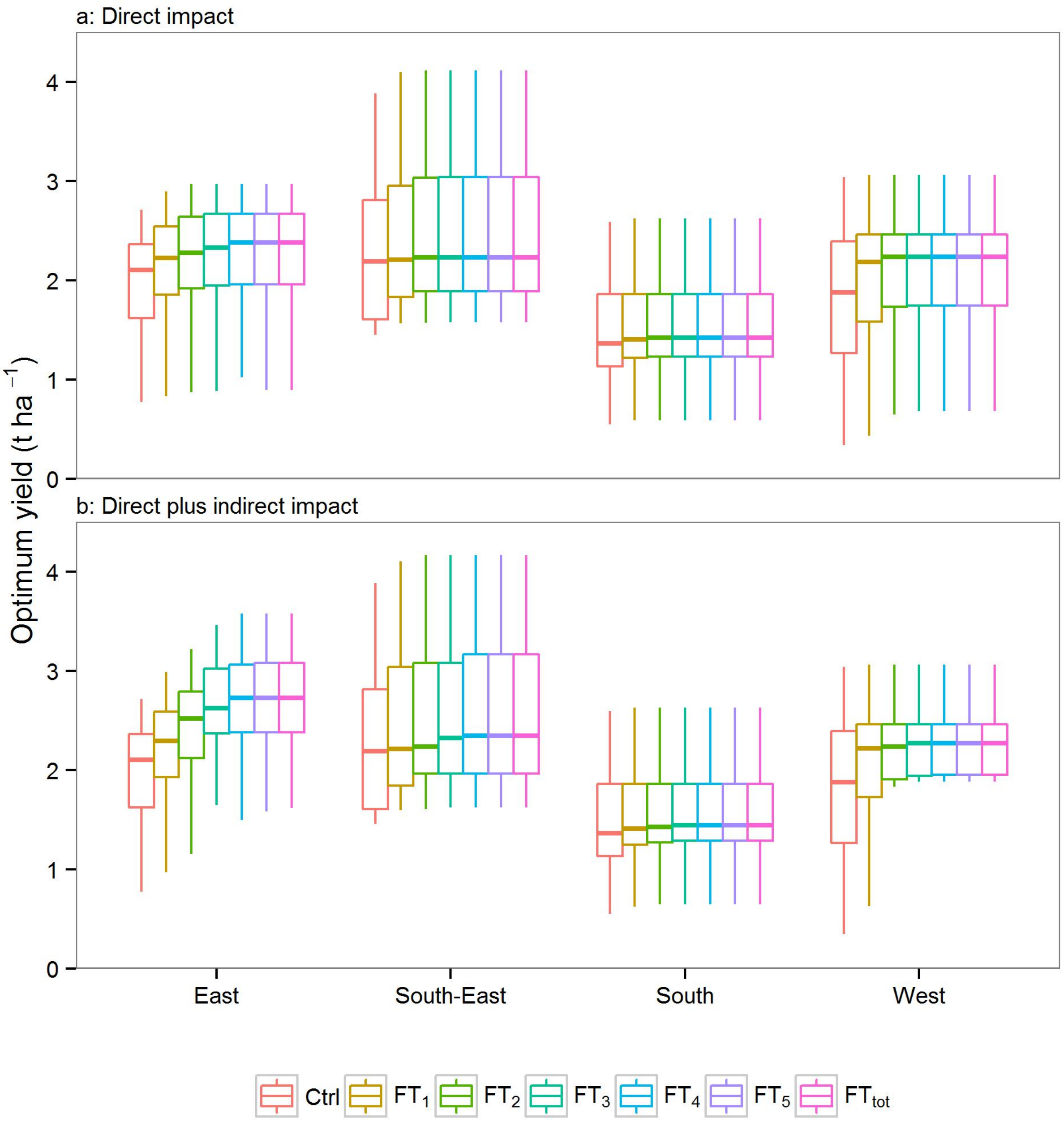

Fig. S8 Direct (a) and direct plus indirect (b) frost impact on yield in each region for a **late-maturing** cultivar with an estimated frost tolerance to 0°C (Control), and for virtual genotypes of the same phenology with improved frost tolerance to -1°C (FT_1), -2°C (FT_2), -3°C (FT_3), -4°C (FT_4), -5°C (FT_5) or total frost tolerance (FT_{tot}). Direct impact refers to yield gain of wheat with improved tolerance, which were sown at the optimum sowing time of current cultivars (Ctrl). Direct plus indirect impact refers to yield gain improved-tolerance crops sown at the optimum sowing time that is specific to each genotype with respect to its levels of frost tolerance. Simulated yield for 60 locations (Fig. 1) from 1957 to 2013.

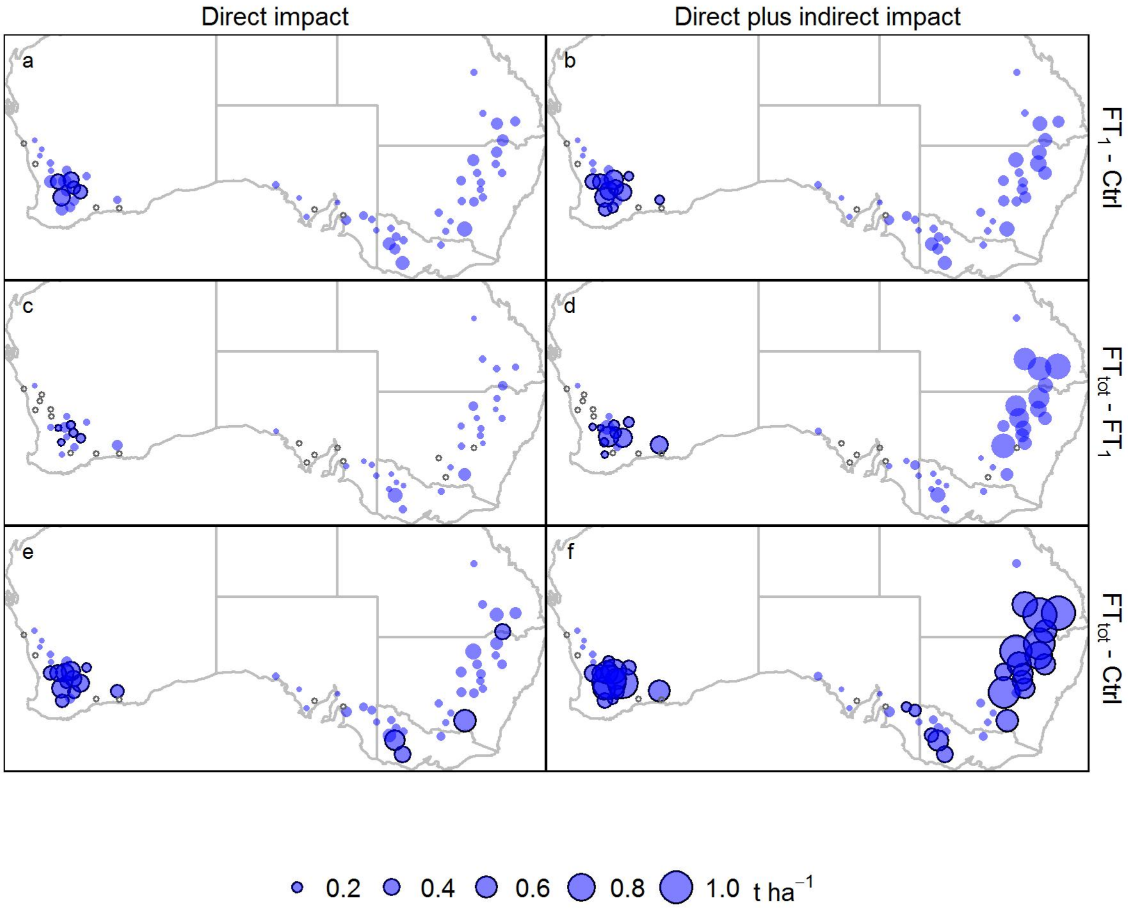

Fig. S9 Simulated mean yield advantage of **early-maturing** cultivars when (i) increasing the frost tolerance to -1°C (i.e. FT_1 - Ctrl; a and b, top maps), (ii) considering the additional yield gain achieved with a total frost tolerance (i.e. FT_{tot} compared to -1°C tolerance, i.e. FT_{tot} - FT_1; c and d, middle), and (iii) looking at the total yield advantage between total tolerance and the current level (FT_{tot} - Ctrl; e and f, bottom). Simulations were done at 60 locations (Fig. 1) for sowing at the optimum sowing date for the current level of tolerance (0°C control; Direct impact; a, c and e, left) or at the optimum sowing date specific to each frost-damage threshold level (Direct plus indirect impact; b, d and f, right). The size of the circle indicates the average yield increase (t ha^{-1}) for 1957-2013. The open small circles indicate the sites where no yield advantage was simulated and the black edges around blue circles indicate that yield advantage was significant ($P < 0.05$).

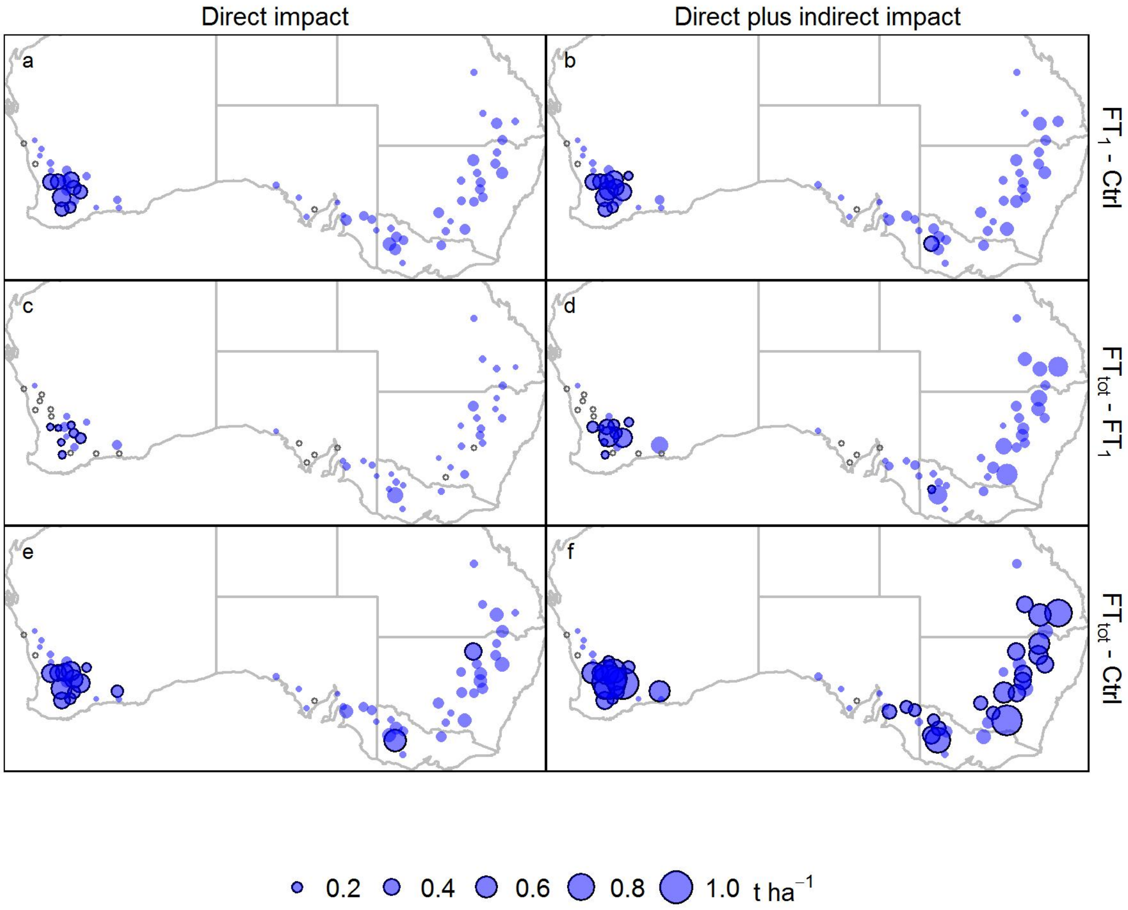

Fig. S10 Simulated mean yield advantage of **late-maturing** cultivars when (i) increasing the frost tolerance to -1°C (i.e. FT_1 - Ctrl; a and b, top maps), (ii) considering the additional yield gain achieved with a total frost tolerance (i.e. FT_{tot} compared to -1°C tolerance, i.e. FT_{tot} - FT_1; c and d, middle), and (iii) looking at the total yield advantage between total tolerance and the current level (FT_{tot} - Ctrl; e and f, bottom). Simulations were done at 60 locations (Fig. 1) for sowing at the optimum sowing date for the current level of tolerance (0°C control; Direct impact; a, c and e, left) or at the optimum sowing date specific to each frost-damage threshold level (Direct plus indirect impact; b, d and f, right). The size of the circle indicates the average yield increase (t ha^{-1}) for 1957-2013. The open small circles indicate the sites where no yield advantage was simulated and the black edges around blue circles indicate that yield advantage was significant ($P < 0.05$).

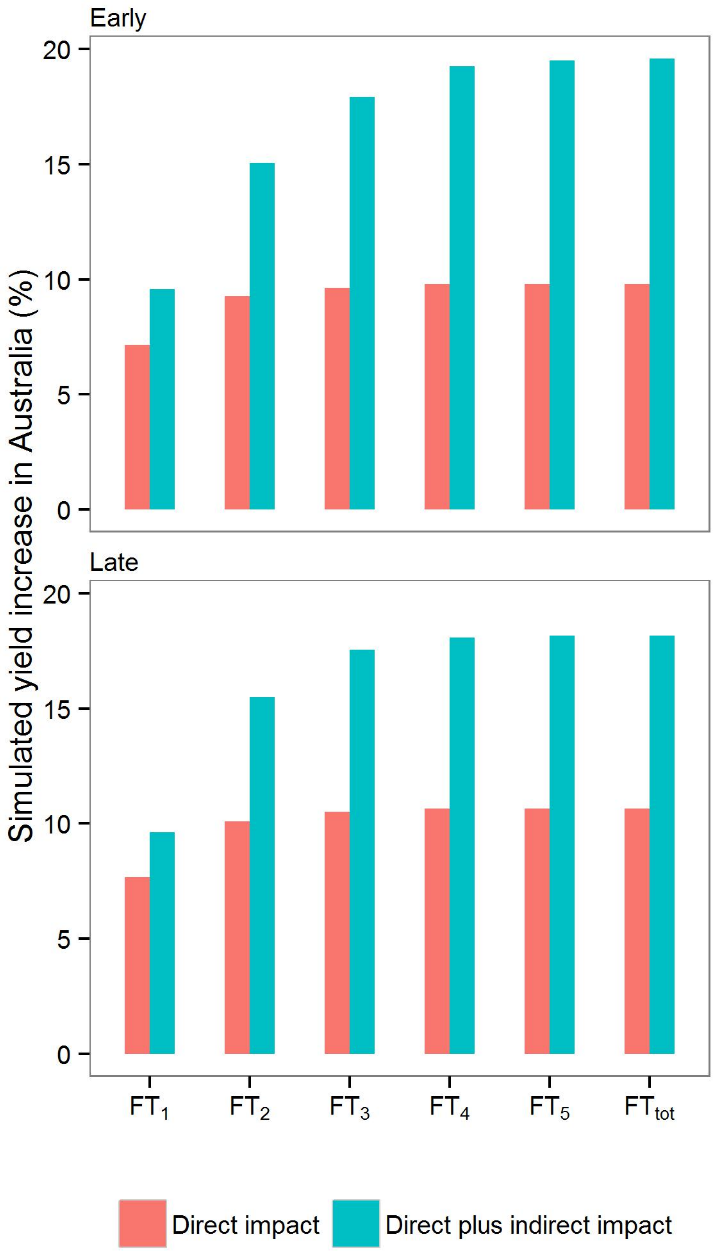

Fig. S11 National yield advantage of reduced damage threshold temperatures of -1°C (FT_1) to total frost tolerance (FT_{tot}) for a **early- and late-maturing cultivar**. Yield advantage was considered when changing either the frost damage threshold temperatures (direct impact; red) or both the frost damage threshold temperatures and the management (direct plus indirect effect; blue). Yield was significantly increased when changing the frost damage threshold temperatures ($FT_{1\text{-}tot}$, $P < 0.05$). Data correspond to average over the 60 locations for simulations for all seasons from 1957 to 2013.

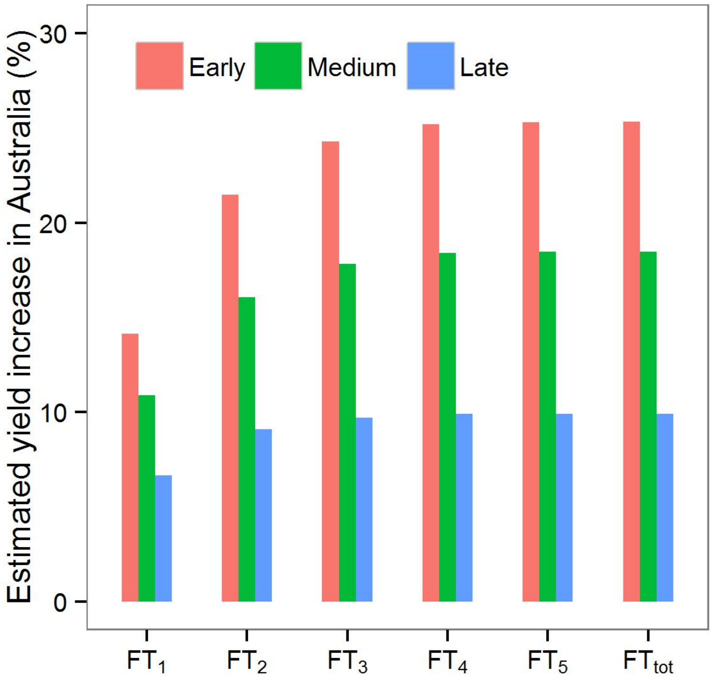

Fig. S12 National ratio of yield advantage for crops **sown over the whole sowing windows** (Table S1) for virtual genotypes of reduced damage threshold temperatures of -1°C (FT_1) to total frost tolerance (FT_{tot}) for a **early-, mid- and late-maturing cultivar**. For each maturity type, yields were significantly increased when changing the frost damage threshold temperatures ($FT_{1\text{-}tot}$, $P < 0.05$). Data correspond to average over the 60 locations for simulations for all seasons from 1957 to 2013.

Reference

Chenu, K., R. Deihimfard, and S. C. Chapman. 2013. Large-Scale Characterization of Drought Pattern: A Continent-Wide Modelling Approach Applied to the Australian Wheatbelt – Spatial and Temporal Trends. **New Phytologist** 198 (3): 801–20.
